# Insights into the Therapeutic Targets and Molecular Mechanisms of *Eruca sativa* Against Colorectal Cancer: An Integrated Approach Combining Network Pharmacology, Molecular Docking and Dynamics Simulation

**DOI:** 10.3390/ph18040453

**Published:** 2025-03-24

**Authors:** Humera Banu, Eyad Al-Shammari, Syed Shahanawaz, Faizul Azam, Mitesh Patel, Naif Abdulrahman Alarifi, Md Faruque Ahmad, Mohd Adnan, Syed Amir Ashraf

**Affiliations:** 1 Department of Clinical Nutrition, College of Applied Medical Sciences, University of Ha’il, Ha’il P.O. Box 2440, Saudi Arabia; humerabanu14@gmail.com (H.B.);; 2 Department of Physiotherapy, College of Applied Medical Sciences, University of Ha’il, Ha’il P.O. Box 2440, Saudi Arabia; 3 Department of Medicinal Chemistry and Pharmacognosy, College of Pharmacy, Qassim University, Buraydah 51452, Saudi Arabia; f.azam@qu.edu.sa; 4 Research and Development Cell, Parul University, Vadodara 391760, Gujarat, India; patelmeet15@gmail.com; 5 Department of Biotechnology, Parul Institute of Applied Sciences, Parul University, Vadodara 391760, Gujarat, India; 6 Senior Specialist Laboratory, Armed Forces Medical Service, Riyadh 13245, Saudi Arabia; 7 Department of Clinical Nutrition, College of Nursing and Health Sciences, Jazan University, Jazan 45142, Saudi Arabia; 8 Department of Biology, College of Science, University of Ha’il, Ha’il P.O. Box 2440, Saudi Arabia; drmohdadnan@gmail.com

**Keywords:** argula, cancer, computational biology, erucin, nutraceuticals, quercetin

## Abstract

**Background/Objectives:** This study presents a novel and comprehensive investigation into the anti-colorectal cancer (CRC) effects and underlying mechanisms of *Eruca sativa* (*E. sativa*) using an integrated approach combining network pharmacology, molecular docking and molecular dynamics simulation. **Methods:** Using an integrated approach, six bioactive compounds and 40 potential targets were identified. A compound–target network was constructed, and enrichment analysis was performed to explore the key pathways influenced by *E. sativa*. Molecular docking analysis was used to evaluate the binding interactions between the identified compounds and key CRC-related targets (AKT1, PGR, MMP9, and PTGS2). Furthermore, molecular dynamics simulation was utilized to confirm the stability and reliability of these interactions. **Results:** The study found that *E. sativa* exhibits strong anticancer potential, particularly through major compounds such as β-ionone, 1-octanol, isorhamnetin, 2-hexenal, propionic acid, and quercetin. Molecular docking revealed favorable binding interactions between these compounds and key CRC targets, with quercetin and isorhamnetin showing the highest binding affinities. Additionally, molecular dynamics simulations validated the stability of these interactions, reinforcing their therapeutic relevance. **Conclusions:** This study provides valuable insights into the pharmacological mechanisms of *E. sativa* against CRC, highlighting its potential as a natural anticancer agent. These findings pave the way for future clinical studies to validate the efficacy and safety of E. sativa and its bioactive compounds, potentially contributing to the development of novel, plant-based therapeutic strategies for CRC treatment.

## 1. Introduction

As one of the most common and lethal malignancies in the world, colorectal cancer (CRC) accounted for 1.9 million new cases and a huge 930,000 deaths by 2020 [1]. While there has been some progress in the diagnosis and treatment of CRC, the prognosis for those patients who have advanced or metastatic disease remains poor despite these advances [2,3,4]. The current treatment options available for advanced stages of CRC often involve chemotherapy and targeted therapies [5]. However, these treatments are often associated with adverse side effects, resistance, and limited efficacy in certain CRC subtypes [6]. Despite advances in the understanding of CRC pathogenesis, significant knowledge gaps remain regarding the precise molecular mechanisms underlying its development and progression. Current therapeutic strategies are primarily focused on targeting individual proteins, such as EGFR, KRAS, and VEGF, but these approaches often fail to address the complex multifactorial nature of the disease [7]. A major challenge in CRC treatment is the heterogeneity of the tumor microenvironment, which includes cancer cells, stromal cells, immune cells, and extracellular matrix components. This complexity often limits the efficacy of conventional therapies and necessitates the development of more specific, multitargeted therapeutic strategies [8]. Moreover, resistance to chemotherapy and targeted agents remains a critical issue, making the need for alternative natural therapies more urgent [9].

As a result, there is an urgent need to search for novel and highly effective therapeutic strategies for the treatment of CRC. Often referred to as rocket salad or arugula, *Eruca sativa* Mill. is an important medicinal herb belonging to the family Brassicaceae. In several parts of the world, especially in the Mediterranean region, it has been used for a variety of purposes, including as a food and medicinal plant [10,11,12,13]. As a result of the presence of multiple bioactive compounds such as glucosinolates, flavonoids, carotenoids, phenolic acids, and terpenoids in *E. sativa*, it is believed to exhibit anticancer, anti-inflammatory, antioxidant, antimicrobial, and immunomodulatory properties [14,15,16]. It has been reported recently that *E. sativa* has the potential to be an anticancer herb that can be used against colorectal cancer [17,18]. Glucosinolates, a prominent group of phytochemicals found in *E. sativa*, are known for their potential anticancer properties. They can be hydrolyzed into bioactive compounds that exhibit cytotoxic effects on cancer cells and may inhibit tumor growth [19]. Additionally, flavonoids such as kaempferol and quercetin, which are abundant in *E. sativa*, have been shown to possess strong antioxidant properties, helping to mitigate oxidative stress and reduce the risk of chronic diseases [20,21]. The antioxidant activity of these compounds is crucial as it aids in the protection of cellular components from damage caused by free radicals [22]. Moreover, *E. sativa* has been reported to exhibit various pharmacological activities, including antibacterial, antidiabetic, antihypertensive, and antiplatelet effects. For instance, studies have demonstrated that extracts from *E. sativa* can inhibit platelet aggregation, suggesting a potential role in cardiovascular health [20,23]. The plant’s oil has also shown promise in improving lipid profiles and protecting against oxidative damage in various experimental models [22,24]. Furthermore, the seeds of *E. sativa* have been linked to enhanced reproductive health, with evidence indicating improvements in semen quality and spermatogenesis in animal studies [20,25].

However, the mechanisms by which *E. sativa* exerts its anticancer effects against CRC remain poorly understood, and its potential to target multiple signaling pathways in CRC has not been fully explored. Limited research has focused on its molecular targets, and the lack of systematic approaches to investigate its effects at the network level has hindered its clinical application. This study seeks to address these gaps by applying an integrated approach combining network pharmacology, molecular docking, and molecular dynamics simulations to identify the key targets and underlying molecular mechanisms of *E. sativa* in CRC. By combining computational and experimental methods, this study aims to provide a deeper understanding of how *E. sativa* interacts with multiple cellular pathways, such as those involved in cell proliferation, apoptosis, inflammation, and metastasis, offering new insights into its therapeutic potential for CRC. Furthermore, in comparison to other plants with known anticancer properties, such as *Curcuma longa* (turmeric), *Withania somnifera* (ashwagandha), and *Camellia sinensis* (green tea), *E. sativa* offers a distinctive chemical composition and an underexplored potential for cancer therapy [26,27,28]. While these other plants have been widely studied for their individual bioactive compounds, *E. sativa* remains relatively less explored despite early evidence suggesting that its phytochemicals could be potent anticancer agents [20,21]. This research gap presents a unique opportunity to further investigate the therapeutic potential of *E. sativa* and explore its efficacy as a novel cancer treatment. Thus, this study aims to contribute to the expanding body of literature on *E. sativa*, providing insights into its bioactive compounds and their molecular interactions with key cancer-related targets.

A promising approach in drug development is network pharmacology, which combines systems biology, bioinformatics, and pharmacology to investigate how drugs interact with biological systems and the complex interactions between them [29,30,31]. In order to predict the properties of drug–target complexes, such as their binding modes and stability, molecular docking and molecular dynamics simulations can be used as computational tools [32,33]. Natural products can be analyzed in this way in order to gain valuable insights into their molecular mechanisms and pharmacological effects. By utilizing network pharmacology, molecular docking, and MD simulations, this study is aimed at examining the potential anti-CRC effects of *E. sativa* on cancer cells. A series of active compounds and potential targets of *E. sativa* have been identified, and their interactions and functions have been analyzed. To evaluate the binding affinity and stability of the selected compounds and targets, we also performed molecular docking and MD simulations in order to look at the binding affinity and stability. As a result of our investigation, we may gain a better understanding of the pharmacological mechanism by which *E. sativa* suppresses CRC and develop new avenues for its development as a natural anticancer agent in the future.

## 2. Results

### 2.1. Screening of Active Compounds and Targets

In the IMPPAT database, a total of 104 active components have been identified as being present in *E. sativa* (Table S1). To identify the most promising bioactive compounds, a multi-step selection process was employed. First, a virtual screening approach was performed on all the constituents of *E. sativa*, considering their oral bioavailability (OB) and drug-likeness (DL). These parameters are critical in determining the absorption, distribution, metabolism, and excretion (ADME) properties of potential therapeutic agents. The criteria for inclusion in the study required that a compound must meet a drug-likeness (DL) value greater than or equal to 0.18, which indicates the compound’s potential for favorable pharmacokinetics and chemical properties suitable for drug development. Additionally, an oral bioavailability (OB) greater than 30% ensures the compound’s effectiveness upon oral administration. Drug-likeness was assessed using the Tanimoto similarity coefficient, which compares the molecular descriptors of a compound to a reference drug database, such as DrugBank. The DL score is calculated using the formula:

$$DL=\frac{A\cdot B}{{\left|A\right|}^{2}+{\left|B\right|}^{2}-A\cdot B}$$

where A represents the molecular properties of the compound, and B represents the properties of known drugs. A threshold of DL ≥ 0.18 was applied to retain compounds with structural and physicochemical characteristics similar to known drugs, increasing their likelihood of successful development. OB was predicted using a machine-learning-based OB-RF model (Random Forest regression model) trained on a dataset of FDA-approved drugs. This model integrates molecular descriptors such as molecular weight, lipophilicity (logP), hydrogen bond donors/acceptors, and rotatable bonds to estimate the fraction of the administered drug reaching the systemic circulation.

**Figure 1:**
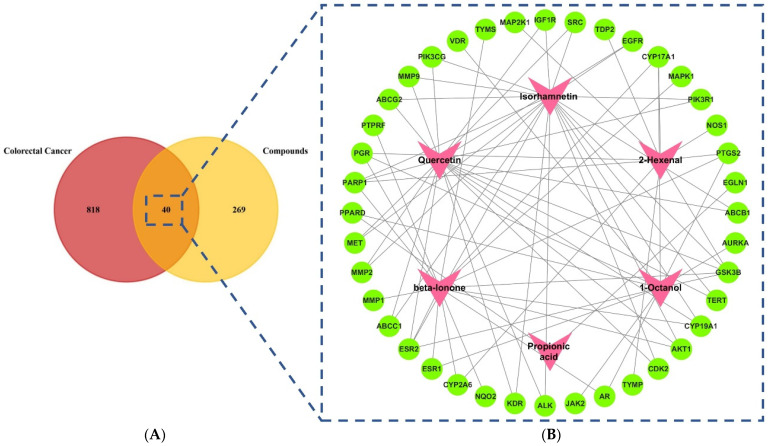
(**A**). Venn diagram illustrating the overlap between the targets of bioactive compounds of *E. sativa* and colorectal cancer targets (**B**). Network representation depicting the interactions among the active components and shared targets identified from *E. sativa*. The pink triangle-shaped nodes represent the active compounds, while the green circular nodes represent the protein targets. The black lines connecting the nodes illustrate the interactions between the compounds and their corresponding targets within the network.

Compounds that did not meet these thresholds for DL and OB were excluded from further analysis. Following this screening, six bioactive compounds, namely beta-ionone, 1-octanol, isorhamnetin, 2-hexenal, propionic acid, and quercetin were selected for further investigation due to their promising ADME properties, favorable drug-likeness and high oral bioavailability. A further investigation revealed that, following the removal of duplications from the SwissTargetPrediction webserver, we were able to find 309 genes that could be the targets of the six active ingredients. A total of 858 genes were identified to be linked to colon cancer based on data downloaded from GeneCards, OMIM, and DisGeNET databases after the duplications were removed in order to identify the most promising drug targets. After that, Venn diagrams were used as a way of determining the common targets for both colon cancer and its compound-linked genes related to the *E. sativa* plant. A total of 40 genes were identified in *E. sativa* that could aid in the fight against colon cancer, and they were identified as key targets in the process (Figure 1).

### 2.2. Compounds–Target Network Construction

**Figure 2:**
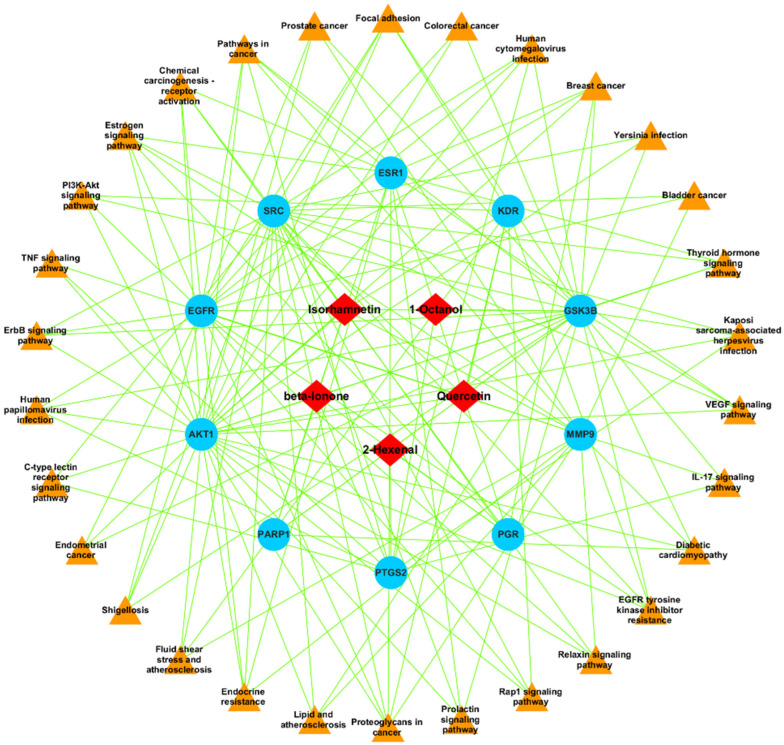
Network representation illustrating the interconnections among active components, their hub targets, and associated signaling pathways in *E. sativa*. The red rhombus-shaped nodes represent the active compounds, the blue circular nodes represent the hub protein targets, and the orange triangle-shaped nodes represent the signaling pathways. The green lines indicate the interactions and connections among the compounds, targets, and pathways within the network.

A total of six active constituents have been identified to be satisfactory from the plant *E. sativa*. To construct the active compound-targeted genes-connected pathway network diagram, a total of six active compounds were used, along with 40 key targets. It has been determined that many of these active compounds have multiple targets, which indicates that when it comes to *E. sativa* being used as an anticancer agent, a synergistic effect will be induced over multiple targets. This was then followed by the assessment of the degree to which these six compounds were connected to the compound-targeted genes-connected pathways network (Figure 2). In addition, all ten hub genes were chosen to be docked into each of the six compounds and one standard drug (camptosar) in order to perform this docking analysis.

### 2.3. PPI Network Construction

**Figure 3:**
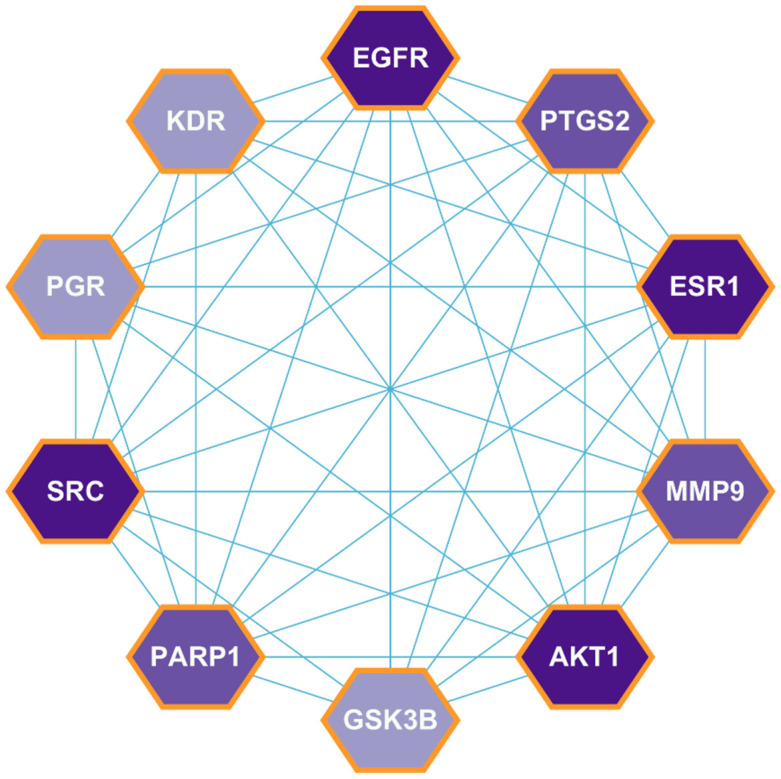
Hub genes network illustrating protein interactions. The proteins are represented by hexagonal-shaped nodes, with a gradation of colors indicating varying degrees of significance. Cyan-color lines represent the interconnections among the proteins, highlighting their interactions within the network.

The 40 genes that overlapped were uploaded to the STRING database to build a PPI network. A PPI network illustrates the interaction between different targets during the development of a disease by showing how they work together. A node and its connections show how these targets are interconnected. The cytohubba plugin of Cytoscape was used later on to find the top 10 hub genes based on the degree method as implemented in cytohubba. The hub genes were identified based on their centrality measures, including degree centrality, betweenness centrality, and closeness centrality. Degree centrality indicates the number of direct connections a node (gene) has in the network, representing its biological significance. Betweenness centrality measures the role of genes in connecting different network components, indicating its regulatory potential. Finally, closeness centrality reflects how efficiently a gene interacts with others in the network, influencing multiple pathways. Based on these parameters, the top hub genes identified were EGFR (32), AKT1 (32), SRC (27), ESR1 (27), PARP1 (25), MMP9 (25), PTGS2 (24), GSK3B (23), PGR (22), and KDR (22) (Figure 3). These genes are reported to be involved in crucial biological functions such as cell proliferation, apoptosis, DNA repair, inflammation, and angiogenesis, making them key regulators in CRC progression. The highest-degree genes have a high degree of correlation between them, which implies that they are highly connected to each other. The cytohubba plugin in the Cytoscape tool identifies all of these genes as hub targets. In Cytoscape, the CytoNCA plugin was further employed for the analysis of topological parameters like betweenness, closeness, subgroup, and degree of active compounds (Tables S2 and S3).

### 2.4. GO and KEGG Pathway Analysis

**Figure 4:**
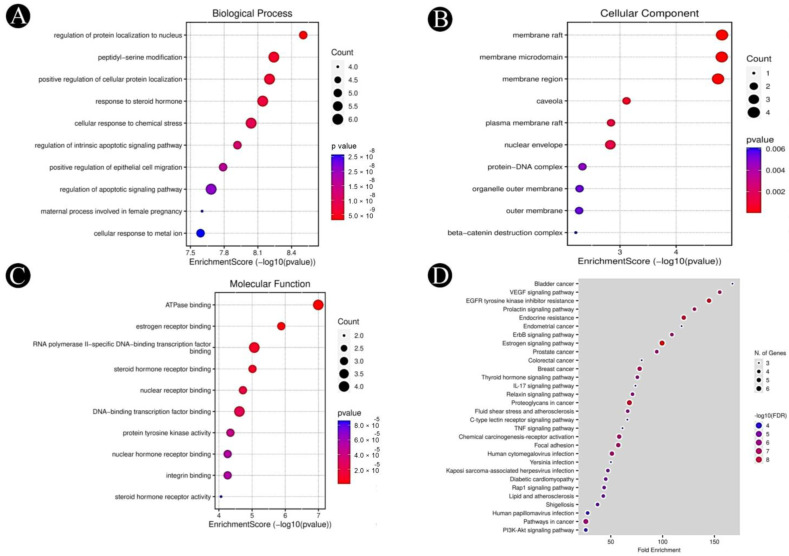
Hub target analysis for GO enrichment and KEGG pathways (*p*-value ≤ 0.05). (**A**). The top 10 biological processes involved (**B**). The top 10 cellular components involved (**C**). The top 10 molecular functions involved, and (**D**). The top 30 KEGG pathways are presented. Color gradients indicate the *p*-values for each term, with darker colors corresponding to lower *p*-values. Dot sizes reflect the number of genes associated with each term, with larger dots indicating a higher gene count.

**Figure 5:**
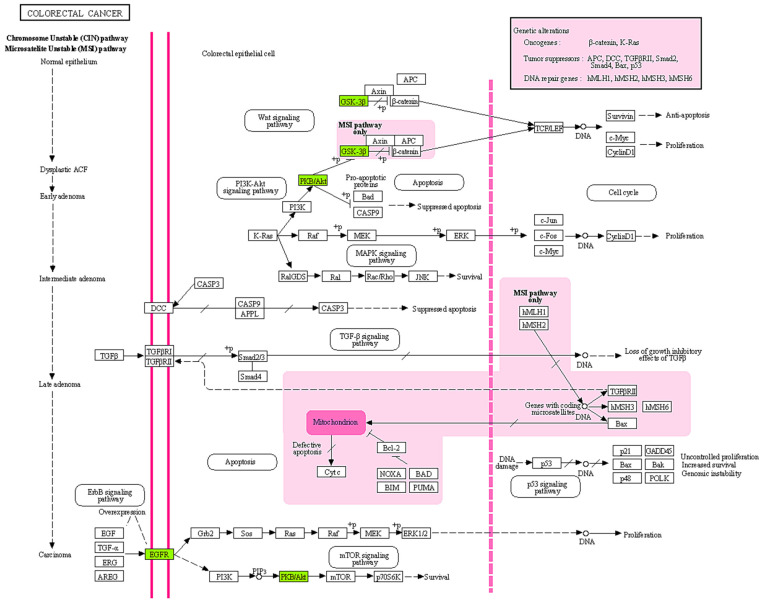
KEGG pathway analysis of hub genes involved in different signaling pathways using the KEGG Pathway Mapper web server.

Functional annotation and enrichment analysis of *E. sativa* targets revealed diverse biological roles. GO and KEGG pathway analyses were performed using SR Plot and ShinyGO (Version 0.80) for ten hub targets. The GO analysis identified 1584 significantly enriched GO terms (*p* < 0.05), categorized into biological processes (1420), cellular components (67), and molecular functions (97). The top-ranked biological processes included protein localization to the nucleus, response to steroid hormones, cellular response to chemical stress, and regulation of apoptosis and epithelial cell migration (Figure 4A–C). Enriched cellular components were primarily associated with membrane structures (raft, microdomain, nuclear envelope, plasma membrane raft) and protein complexes (β-catenin destruction complex). Key molecular functions included ATPase binding, estrogen receptor binding, nuclear receptor binding, and protein tyrosine kinase activity. KEGG pathway enrichment analysis identified 141 significantly associated pathways (*p* < 0.05), including VEGF signaling, EGFR tyrosine kinase inhibitor resistance, estrogen signaling, PI3K–Akt signaling, and pathways related to colorectal cancer (Figure 4D). Notably, the VEGF signaling pathway, crucial in CRC progression, was enriched among the ten hub genes (AKT1, PGR, PTGS2, MMP9, PARP1, GSK3B, SRC, EGFR, ESR1 and KDR). Given its role in angiogenesis, tumor growth, and metastasis, inhibition of VEGF-mediated signaling suggests that *E. sativa* may exert anti-angiogenic and tumor-suppressive effects, highlighting its potential therapeutic relevance in CRC (Figure 5).

### 2.5. Molecular Docking

**Figure 6:**
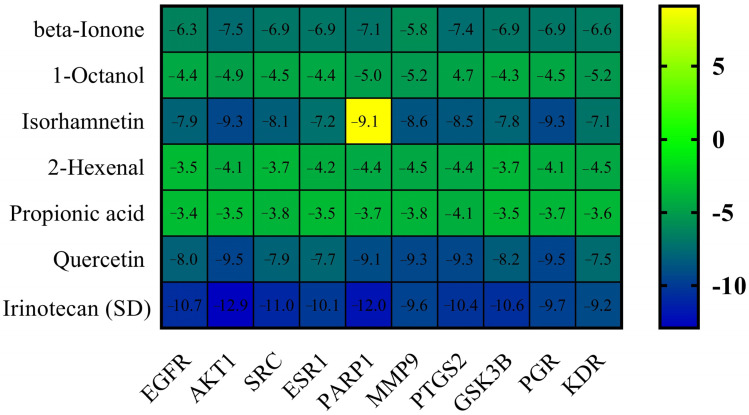
Heatmap illustrating the top binding affinities of the bioactive compounds of *E. sativa* against colorectal cancer targets. The color gradient from yellow to blue indicates an increase in binding energy, with yellow representing the lowest binding affinities and blue representing the highest. The scale bar at the side of the heatmap provides a reference for the binding energy range.

**Figure 7:**
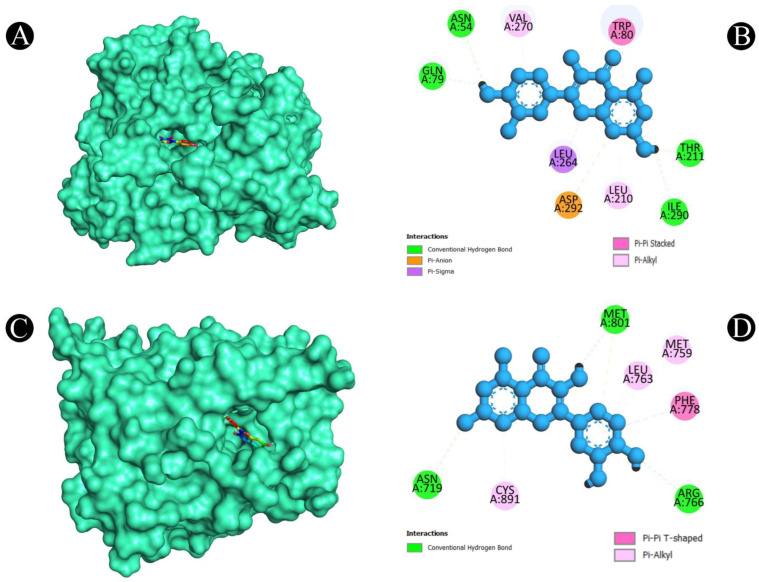
Illustration of the binding interactions between bioactive compounds of *E. sativa* with colorectal cancer targets. (**A**). Three-dimensional hydrophobicity surface representation of quercetin (rainbow color) with the AKT1 protein (cyan color) (**B**). Two-dimensional interactions between quercetin and the AKT1 protein (**C**). Three-dimensional hydrophobicity surface representation of quercetin (rainbow color) with the PGR protein (**D**). Two-dimensional interactions between quercetin and the PGR protein.

**Figure 8:**
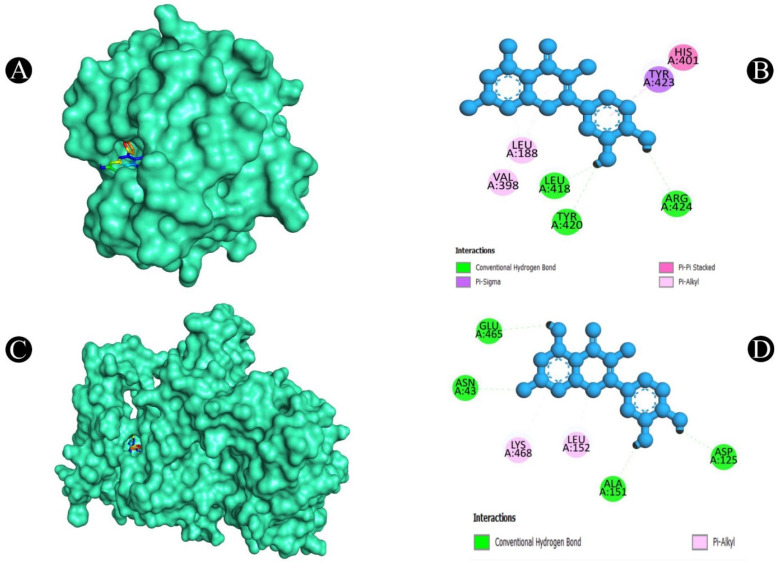
(**A**). Three-dimensional hydrophobicity surface representation of quercetin (rainbow color) with the MMP9 protein (cyan color) (**B**). Two-dimensional interactions between quercetin and the MMP9 protein. (**C**). Three-dimensional hydrophobicity surface representation of quercetin (rainbow color) with the PTGS2 protein (cyan color) (**D**) Two-dimensional interactions between quercetin and the PTGS2 protein.

**Figure 9:**
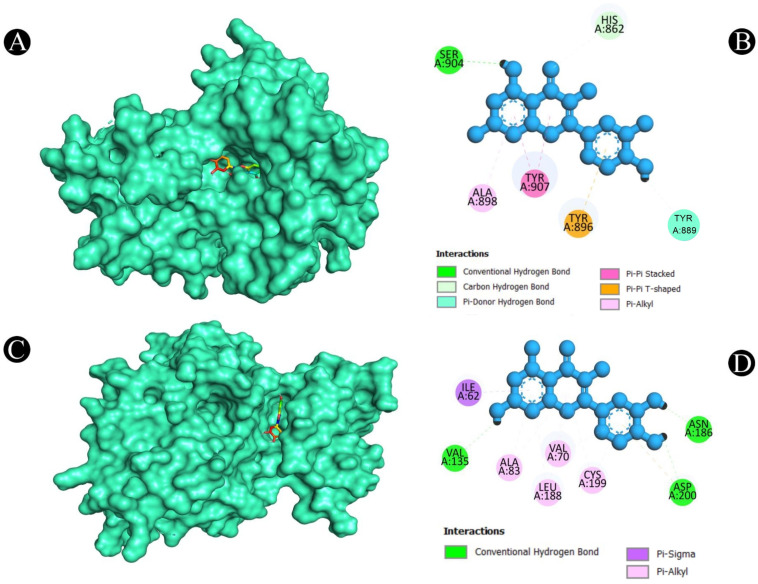
(**A**). Three-dimensional hydrophobicity surface representation of quercetin (rainbow color) with the PARP1 protein (cyan color) (**B**). Two-dimensional interactions between quercetin and the PARP1 protein. (**C**). Three-dimensional hydrophobicity surface representation of quercetin (rainbow color) with the GSK3B protein (cyan color) (**D**) Two-dimensional interactions between quercetin and the GSK3B protein.

**Figure 10:**
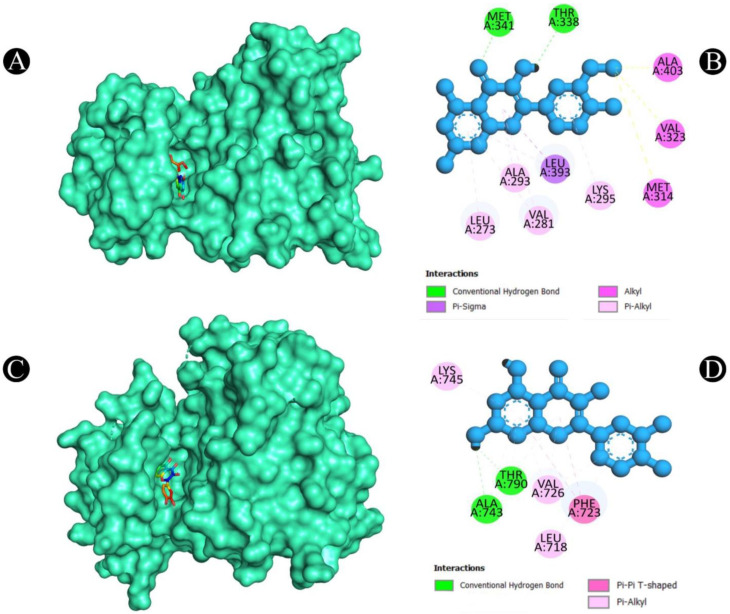
(**A**). Three-dimensional hydrophobicity surface representation of isorhamnetin (rainbow color) with the SRC protein (cyan color) (**B**). Two-dimensional interactions between isorhamnetin and the SRC protein. (**C**). Three-dimensional hydrophobicity surface representation of quercetin (rainbow color) with the EGFR protein (cyan color) (**D**) Two-dimensional interactions between quercetin and the EGFR protein.

**Figure 11:**
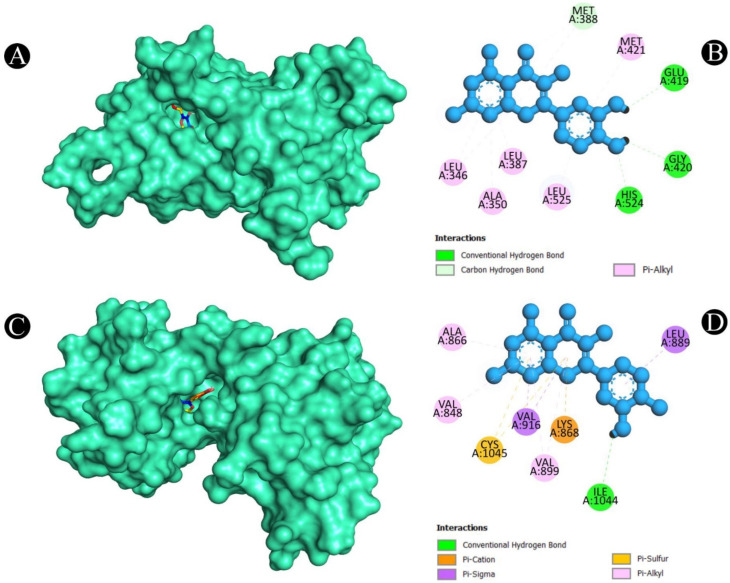
(**A**). Three-dimensional hydrophobicity surface representation of quercetin (rainbow color) with the ESR1 protein (cyan color) (**B**). Two-dimensional interactions between quercetin and the ESR1 protein. (**C**). Three-dimensional hydrophobicity surface representation of quercetin (rainbow color) with the SRC protein (cyan color) (**D**) Two-dimensional interactions between quercetin and the SRC protein.

After a thorough analysis of the PPI network, the top ten hub genes were chosen for molecular docking analysis. A PDB structure for each target protein was selected based on the resolution of the structure. Low numerical values of less than 3 Å indicate an acceptable structural resolution. In the present study, protein structures used among AKT1 had a resolution of 2.80 Å, PGR had a resolution of 1.80 Å, PTGS2 had a resolution of 2.41 Å, MMP9 had a resolution of 2.30 Å, PARP1 had a resolution of 2.06 Å, GSK3B had a resolution of 2.40 Å, SRC had a resolution of 2.10 Å, EGFR had a resolution of 2.80 Å, ESR1 had a resolution of 1.90 Å, and KDR had a resolution of 2.50 Å, respectively. For the purpose of molecular docking, six active ingredients, one standard drug (camptosar), and the top 10 hub targets were selected. The molecular docking analysis revealed that several phytochemical constituents exhibited high affinity toward specific target proteins (Figure 6). Quercetin exhibited strong binding affinity with AKT1 (−9.5 kcal/mol), forming key interactions with functionally significant residues. Notably, it established four conventional hydrogen bonds with ASN54, GLN79, THR211, and ILE290, which are involved in stabilizing the protein’s active conformation. Additionally, a pi–anion bond with ASP292 and a pi–sigma bond with LEU264 may contribute to ligand stability within the binding site. The presence of four pi–pi stacked bonds with TRP80 and two pi–alkyl interactions with LEU210 and VAL270 suggests enhanced hydrophobic stabilization. Similarly, quercetin demonstrated a binding affinity of −9.5 kcal/mol toward PGR, interacting with key residues such as ASN719, ARG766, and MET801 through hydrogen bonding, which may play a role in ligand recognition and receptor activation. A pi–sulfur bond with MET801, a pi–pi T-shaped bond with PHE778, and three pi–alkyl interactions with CYS891, MET759, and LEU763 further support the stability of the ligand within the binding pocket. Against MMP9 (−9.3 kcal/mol), quercetin formed conventional hydrogen bonds with LEU418, TYR420, and ARG424, which are critical residues for substrate binding. Additionally, a pi–sigma bond with TYR423, a pi–pi stacked interaction with HIS401, and three pi–alkyl interactions with LEU188 and VAL398 enhance the ligand’s stability within the catalytic site, potentially influencing enzymatic inhibition. For PTGS2 (−9.3 kcal/mol), the hydrogen bonds formed with ASN43, ALA151, ASP125, and GLU465 indicate potential involvement in modulating enzymatic activity. The two pi–alkyl bonds with LEU152 and LYS468 suggest additional stabilization via hydrophobic interactions. Quercetin also exhibited strong binding with PARP1 (−9.1 kcal/mol), forming a crucial hydrogen bond with SER904, which is known to be involved in NAD^+^ binding. Additional stabilizing interactions include a carbon–hydrogen bond with HIS862, a pi–donor hydrogen bond with TYR889, two pi–pi stacked bonds with TYR907, a pi–pi T-shaped bond with TYR896, and a pi–alkyl interaction with ALA898, which may contribute to potential inhibition of PARP1 activity. In the case of GSK3B (−8.2 kcal/mol), hydrogen bonds with ASN186, ASP200, and VAL135 were observed, with ASP200 also forming a pi–anion bond, which is relevant for ATP binding and kinase activity regulation. The presence of one pi–sigma bond with ILE62 and multiple pi–alkyl interactions with VAL70, ALA83, LEU188, and CYS199 suggests a well-stabilized binding pose within the kinase domain. Against EGFR (−8.0 kcal/mol), quercetin formed two conventional hydrogen bonds with ALA743 and THR790, which are critical for ATP binding and kinase function. A pi–donor hydrogen bond with THR790, two pi–pi T-shaped bonds with PHE723, and seven pi–alkyl interactions (VAL726, ALA743, LYS745, and LEU718) further contribute to ligand stability within the active site, potentially affecting EGFR signaling. For ESR1 (−7.7 kcal/mol), hydrogen bonds with HIS524, GLU419, and GLU420 suggest potential modulation of estrogen receptor activity. Additional stabilizing interactions include a carbon–hydrogen bond with MET388 and multiple pi–alkyl interactions with LEU346, MET388, LEU525, ALA350, LEU387, and MET421, which may enhance receptor–ligand affinity. KDR (−7.5 kcal/mol) exhibited one hydrogen bond with ILE1044, a pi–cation bond with LYS868, and three pi–sigma bonds with LEU889 and VAL916, which are known to play a role in ATP binding. Two pi–sulfur bonds with CYS1045 and four pi–alkyl interactions with VAL899, VAL848, and ALA866 indicate additional ligand stability. Lastly, isorhamnetin showed a notable binding affinity with SRC (−8.1 kcal/mol), forming hydrogen bonds with MET341 and THR338, which are crucial for ATP binding and kinase activation. Additional interactions included a pi–sigma bond with LEU393, three alkyl interactions (ALA403, MET314, and VAL323), and six pi–alkyl interactions (LEU273, VAL281, ALA293, LEU393, and LYS295), suggesting a potential role in SRC inhibition. In both Figure 7, Figure 8, Figure 9, Figure 10 and Figure 11 and Table S4, the best active phytochemical compound of *E. sativa* is shown to occupy the active site in different ways.

### 2.6. MD Simulation Analysis

**Figure 12:**
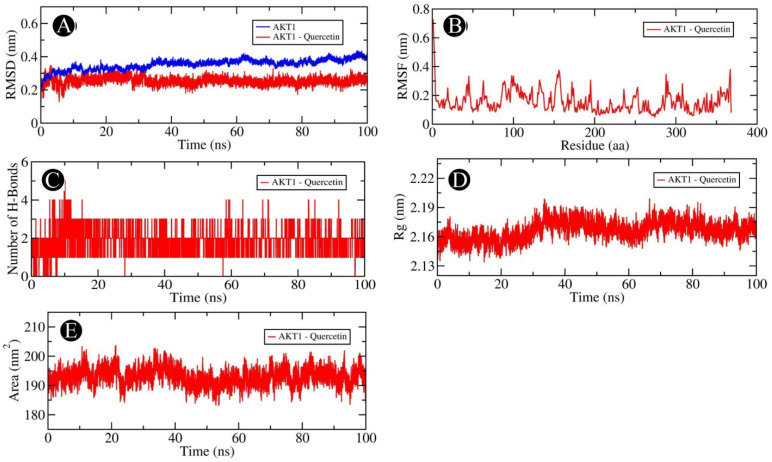
Molecular dynamics simulation analysis of the AKT1 protein and quercetin molecule over time. (**A**) RMSD analysis of AKT1 with and without quercetin binding, (**B**) RMSF analysis of the AKT1–quercetin complex, (**C**) AKT1–quercetin complex intramolecular H-bond time evolution, (**D**) Rg distribution of the AKT1–quercetin complex, and (**E**) SASA plot analysis of the AKT1–quercetin complex.

**Figure 13:**
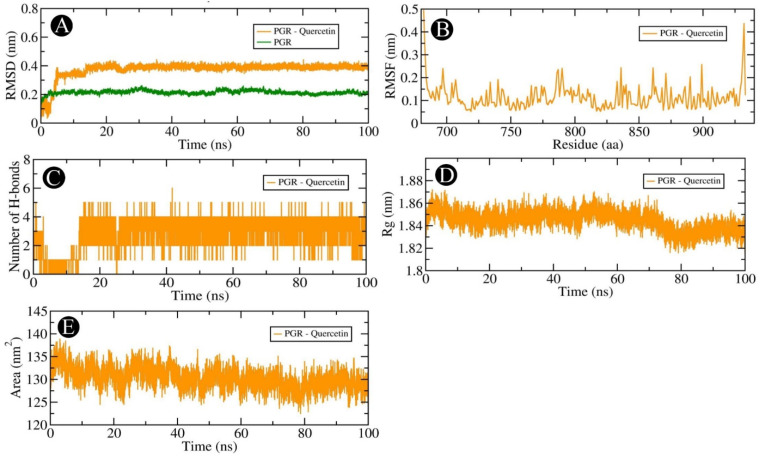
Molecular dynamics simulation analysis of the PGR protein and quercetin molecule over time. (**A**) RMSD analysis of PGR with and without quercetin binding, (**B**) RMSF analysis of the PGR–quercetin complex, (**C**) PGR–quercetin complex intramolecular H-bond time evolution, (**D**) Rg distribution of the PGR–quercetin complex, (**E**) SASA plot analysis of the PGR–quercetin complex.

**Figure 14:**
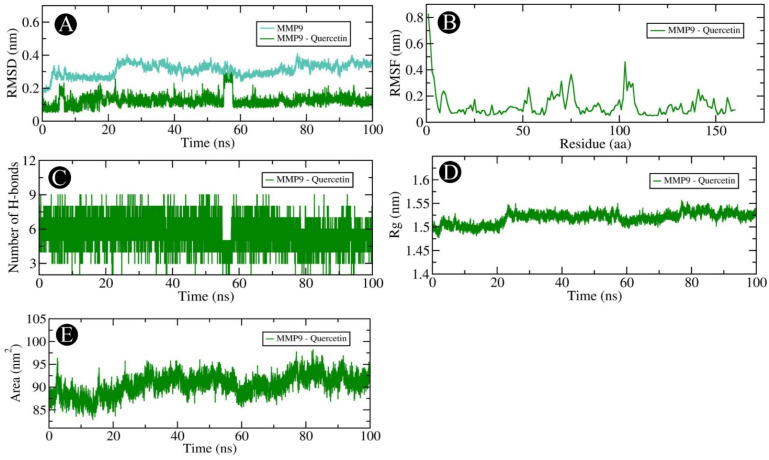
Molecular dynamics simulation analysis of the MMP9 protein and quercetin molecule over time. (**A**) RMSD analysis of MMP9 with and without quercetin binding, (**B**) RMSF analysis of the MMP9–quercetin complex, (**C**) MMP9–quercetin complex intramolecular H-bond time evolution, (**D**) Rg distribution of the MMP9–quercetin complex, and (**E**) SASA plot analysis of the MMP9–quercetin complex.

**Figure 15:**
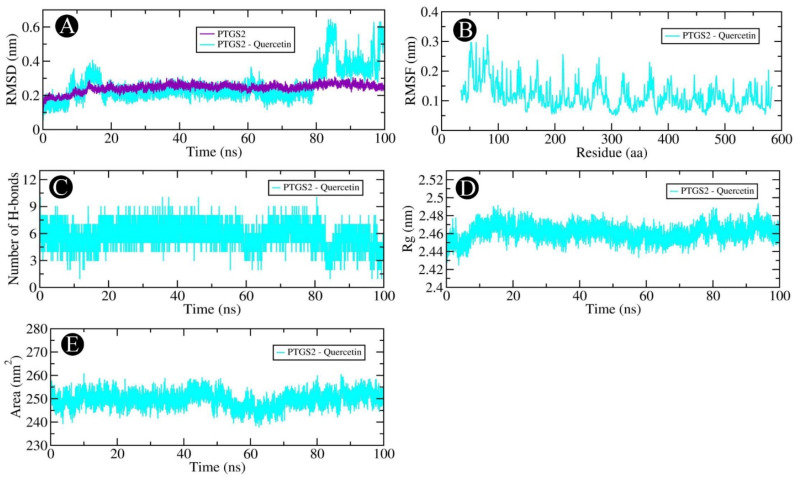
Molecular dynamics simulation analysis of the PTGS2 protein and quercetin molecule over time. (**A**) RMSD analysis of PTGS2 with and without quercetin binding, (**B**) RMSF analysis of the PTGS2–quercetin complex, (**C**) PTGS2–quercetin complex intramolecular H-bond time evolution, (**D**) Rg distribution of the PTGS2–quercetin complex, and (**E**) SASA plot analysis of the PTGS2–quercetin complex.

Our docking-based assessment led us to select AKT1, PGR, PTGS2, and MMP9, which showed very good binding affinity with quercetin for MD studies. In order to determine the dynamics of quercetin in complex with AKT1, PGR, PTGS2, and MMP9 proteins, MD studies were carried out. Using a docked complex of quercetin with AKT1, PGR, PTGS2, and MMP9, we examined the stability and structural flexibility of the ligand–protein complex. In order to perform the analysis, GROMACS (version 2019.4) software was used at 100 ns. The RMSD analysis can be used to determine the deviation in the structure of proteins and ligand–protein complexes by examining the RMSD values. To determine the stability and movement of AKT1, PGR, PTGS2, and MMP9 in the solvent environment, structural deviations of these complexes with quercetin were investigated during the simulation. Based on the results of the simulation, it was observed that the RMSDs of the backbones of AKT1, AKT1–quercetin, PGR, and PGR–quercetin docked complexes, and MMP9, and MMP9–quercetin docked complexes exhibited a stable pattern throughout the simulation run. There was an average RMSD of 0.254 nm for AKT1 and 0.304 nm for the AKT1–quercetin complex, respectively (Figure 12A). The average RMSDs for the PGR and the PGR–quercetin complex were 0.374 nm and 0.217 nm, respectively, according to Figure 13A. The average RMSDs of MMP9 and the MMP9–quercetin complex were 0.213 nm and 0.125 nm, respectively (Figure 14A). There was little fluctuation in the pattern at 80–100 ns due to ligand binding between PTGS2 and quercetin-docked complexes (Figure 15A). There was an average RMSD of 0.258 nm observed for PTGS2 and 0.251 nm observed for PTGS2–quercetin complex, respectively. There were no significant shifts in the RMSD patterns in the distribution of the protein–ligand complex during the simulation, suggesting that during the simulation, there was a strong ligand-binding strength among AKT1, PGR, MMP9, and PTGS2 despite the distribution of RMSD patterns. In terms of protein structure, the RMSF indicates the degree of flexibility of each residue in the protein. There was an average fluctuation of 0.11 nm in AKT1–quercetin (Figure 12B), 0.14 nm in PGR–quercetin (Figure 13B), MMP9–quercetin (Figure 14B), and 0.15 nm with PTGS2–quercetin complex (Figure 15B) during the simulation. There is a remarkable consistency in the way the AKT1–quercetin, PGR–quercetin, MMP9–quercetin, and PTGS2–quercetin interact on the basis of the graph. The stability and integrity of protein structures are determined by the H-bonds within them. It is helpful to examine the time evolution of the formation and breakdown of H-bonds during simulation time in order to obtain a clearer picture of the structural integrity and stability of protein–ligand complexes. Within the docked AKT1, PGR, MMP9, and PTGS2 proteins with quercetin, intermolecular hydrogen bonds are formed, resulting in the stability of the protein and its ligand. An average of three H-bonds maintained the docked complex of AKT1–quercetin (Figure 12C), whereas four H-bonds were maintained by PGR–quercetin (Figure 13C), eight H-bonds were maintained by MMP9–quercetin (Figure 14C) and eight H-bonds were maintained by PTGS2–quercetin (Figure 15C). This simulation was conducted in order to examine their time evolution over the course of the simulation process and determine how it changes over time. In addition to calculating molecular stability, one can also estimate molecular stability from the compactness of a protein molecule. There is a measure of compactness called Rg that is used in MD simulations. In order to examine the tertiary structure of the protein, it is useful to consider the compactness of the protein structure as a parameter. Rg values have been used to determine the degree of compactness between AKT1–quercetin, PGR–quercetin, MMP9–quercetin, and PTGS2–quercetin binding. There is an average Rg value of 2.16 nm for AKT1–quercetin complex (Figure 12D), 1.84 nm for PGR–quercetin complex (Figure 13D), 1.51 nm for MMP9–quercetin complex (Figure 14D) and 2.46 nm for PTGS2–quercetin complex (Figure 15D), respectively. There is no significant change in the compactness of the protein–ligand complex throughout the simulation, as shown by the Rg plot. SASA refers to the area of a protein molecule that is accessible to its neighboring solvent when it is attached to a protein molecule. There is a wide use of SASA analysis during simulations in order to examine protein folding or unfolding as well as the structural stability of proteins. There were no significant peaks in SASA values based on the simulation, which indicates quercetin binding was not able to affect the folding of AKT1, PGR, MMP9, and PTGS2. There was an average of 192.96, 130.23, 90.52 and 249.65 nm^2^ for SASA values for AKT1–quercetin (Figure 12E), PGR–quercetin (Figure 13E), MMP9–quercetin (Figure 14E) and PTGS2–quercetin (Figure 15E), respectively. There was no change in the stability of AKT1, PGR, MMP9, and PTG as a result of the binding of their respective ligands to their respective receptors, as shown by SASA values.

## 3. Discussion

The aim of this study was to explore the anti-CRC effects and mechanisms of *E. sativa*, a leafy vegetable with various bioactive compounds present within it, using network pharmacology, molecular docking, and molecular dynamics simulations. We identified six phytochemicals and 40 potential targets of *E. sativa* and constructed a compound–target network to visualize their interactions. Additionally, KEGG and GO pathway enrichment analyses were carried out in order to gain insight into the biological processes that are involved in anti-CRC action of *E. sativa*. Among the identified pathways, the VEGF signaling pathway plays a crucial role in promoting angiogenesis, tumor growth and metastasis in CRC by enhancing vascular permeability and endothelial cell proliferation [34]. The EGFR tyrosine kinase inhibitor resistance pathway contributes to therapy resistance by activating alternative survival pathways, leading to poor treatment outcomes [35]. The prolactin signaling pathway regulates cell proliferation, apoptosis and inflammation, influencing CRC progression [36]. The ErbB signaling pathway is involved in cell proliferation and survival and is frequently dysregulated in CRC, making it a potential therapeutic target [37]. The estrogen signaling pathway has been shown to modulate CRC risk and progression, with protective effects observed in some cases [38]. The colorectal cancer pathway encompasses key genetic and molecular alterations that drive CRC initiation and progression, providing insights into potential intervention points. The thyroid hormone signaling pathway influences metabolism and proliferation, which can impact CRC development [39]. The IL-17 signaling pathway is associated with inflammation, immune evasion, and tumor progression, making it a crucial factor in the tumor microenvironment [40]. The relaxin signaling pathway regulates extracellular matrix remodeling, facilitating metastasis [41]. The C-type lectin receptor signaling pathway modulates immune response and tumor-associated inflammation, influencing cancer progression [42]. The Rap1 signaling pathway plays a key role in cell adhesion, migration, and invasion, which are essential processes in CRC metastasis [43]. The pathways in the cancer category integrate multiple oncogenic signaling cascades, highlighting the complex interplay of different molecular mechanisms in CRC. Lastly, the PI3K–Akt signaling pathway regulates cell survival, proliferation, and metabolism, playing a central role in CRC progression and resistance to apoptosis. Understanding these pathways provides valuable insights into the molecular mechanisms of CRC and potential therapeutic strategies [44].

The molecular docking analysis revealed significant binding affinities between the selected phytochemicals (beta-ionone, 1-octanol, isorhamnetin, 2-hexenal, propionic acid, and quercetin) and the top ten hub target proteins, which could potentially translate into therapeutic benefits for various disease conditions. The key binding interactions, such as hydrogen bonds, pi interactions, and alkyl interactions, suggest the strength and specificity of these interactions, making these compounds promising candidates for further exploration [45]. The modulation of pathways involved in cancer metastasis (MMP9), inflammation (PTGS2), cell survival (AKT1), and DNA repair (PARP1) suggests that these compounds could serve as multitargeted therapies, providing an advantage over single-target drugs [46]. The presence of key interactions like hydrogen bonding, pi stacking, and alkyl interactions further strengthens that these compounds can bind with high specificity and affinity, promoting their use in disease management. However, future in vitro and in vivo studies are essential to validate these findings and to assess the pharmacokinetic and pharmacodynamic properties of these compounds for clinical application. To further evaluate the stability, flexibility, and reliability of the protein–ligand complexes, we performed MD simulations and analyzed key structural parameters, including RMSD, RMSF, Rg, SASA, and H-bond analysis. These analyses provide insights into the conformational stability and dynamic behavior of the protein–ligand interactions, which are crucial for assessing their therapeutic potential. The combined analysis of RMSD, RMSF, Rg, SASA, and H-bond provides strong evidence for the stability and reliability of the studied phytochemicals as potential therapeutic agents [47]. The stable RMSD values confirm structural integrity; the low RMSF values indicate minimal flexibility in the binding regions; the consistent Rg values demonstrate compactness, and the controlled SASA fluctuations reflect natural structural dynamics. Additionally, the sustained hydrogen bond interactions further reinforce the strength and specificity of ligand binding. The ability of these compounds to maintain strong and stable interactions with key target proteins suggests their potential efficacy in modulating disease-related pathways [48,49,50]. Moreover, the compact and stable conformations observed in the simulations indicate that these phytochemicals may exhibit favorable binding affinities in physiological conditions, further strengthening their therapeutic potential. These findings support the molecular docking results by providing dynamic validation of the ligand–protein interactions, ensuring that the identified compounds are not only promising in docking studies but also stable over time, making them viable candidates for further experimental and clinical exploration [47,48].

Camptosar (irinotecan) is a chemotherapy drug commonly used in the treatment of various cancers, including CRC, small-cell lung cancer, and ovarian cancer. It is a topoisomerase I inhibitor that works by interfering with the DNA replication process, thereby preventing cancer cell division and promoting cell death. Camptosar is often administered in combination with other chemotherapeutic agents to enhance its therapeutic efficacy [51]. Despite its clinical success, the drug is associated with several side effects, including gastrointestinal toxicity, myelosuppression, and potential resistance development, which may limit its long-term effectiveness [51]. In this study, we compared the binding energies and stability of Camptosar with several bioactive compounds from *E. sativa* in an effort to identify alternative therapies that might offer similar or improved therapeutic benefits with potentially fewer side effects. Camptosar exhibited binding energies in the molecular docking analysis ranging from −9.2 to −12.9 kcal/mol against the hub genes, demonstrating strong interactions with key target proteins. Similarly, various phytochemicals from *E. sativa* also demonstrated comparable binding affinities, suggesting their potential as therapeutic alternatives with possibly improved side effect profiles. By providing this comparative analysis, our aim was to highlight the therapeutic potential of *E. sativa* compounds as promising candidates for further development in cancer treatment, potentially offering a more targeted approach with fewer adverse effects than current treatments like Camptosar.

Our network pharmacology analysis showed that *E. sativa* could modulate multiple targets and signaling pathways related to CRC, such as VEGF, EGFR tyrosine kinase inhibitor resistance, prolactin, ErbB, estrogen, colorectal cancer, thyroid hormone, IL-17, relaxin, C-type lectin receptor, Rap1, pathways in cancer and PI3K–Akt signaling pathways. Based on previous studies, *E. sativa* and its compounds demonstrated anticancer activity in multiple cancer models, including colorectal cancer, which is consistent with the results of the current study [10,11,12]. According to Michael et al. (2011) [52], a 70% ethanolic extract of *E. sativa* has been discovered to exhibit potent anticancer properties against several types of human cancer cell lines, including HepG2 (liver carcinoma), MCF7 (breast carcinoma), HCT116 (colon carcinoma) and Hep2 (larynx carcinoma). The anticancer potential of aerial roots and seed oil extracts of *E. sativa* was evaluated by Khoobchandani et al. (2011) [53] against melanoma cells. As a result of the large amount of isothiocyanates present in the seed oil, the oil appeared to be highly effective in reducing tumor growth and angiogenesis in mice without causing significant harm. The present study revealed quercetin and isorhamnetin had favorable binding interactions with the four key targets, as indicated by their low docking scores. Quercetin and isorhamnetin, both flavonoids, have garnered attention for their potential anticancer effects, particularly against CRC, as well as their associated toxicity profiles. Quercetin, a widely studied flavonoid found in various fruits and vegetables, has been shown to exert significant anticancer properties through multiple mechanisms, including the induction of apoptosis, cell cycle arrest and modulation of signaling pathways involved in cancer progression [54,55,56]. Specifically, quercetin has been reported to inhibit the proliferation of CRC cells by activating the AMPK signaling pathway, which leads to increased apoptosis and reduced tumor growth [54,55]. Additionally, quercetin has demonstrated the ability to enhance the efficacy of other chemotherapeutic agents, suggesting its potential as an adjunct therapy in cancer treatment [56,57]. Isorhamnetin, a methylated derivative of quercetin, has also shown promising anticancer effects, particularly against human CRC cells. Research indicates that isorhamnetin can inhibit cell proliferation and induce apoptosis in CRC cell lines, such as HCT-116, in a dose-dependent manner [58]. The cytotoxic effects of isorhamnetin are attributed to its ability to modulate various cellular pathways, similar to quercetin, thereby contributing to its potential as a therapeutic agent against CRC [58]. Furthermore, both quercetin and isorhamnetin exhibit antioxidant properties, which may help mitigate oxidative stress associated with cancer progression [59]. Despite their therapeutic potential, the toxicity of quercetin and isorhamnetin remains a concern. While quercetin is generally considered safe at dietary levels, high doses may lead to adverse effects, including gastrointestinal disturbances and interactions with certain medications [60]. Isorhamnetin, although less studied, may also pose risks at elevated doses, necessitating further investigation into its safety profile [61]. The balance between their anticancer efficacy and potential toxicity is crucial for their application in clinical settings, highlighting the need for careful dosing and monitoring in therapeutic contexts. Among the various phytochemicals analyzed through molecular docking analysis in the present study, quercetin was specifically highlighted due to its consistently high docking scores and strong binding interactions with key residues in the active sites of target proteins. Additionally, quercetin has been extensively reported for its pharmacological properties, including antioxidant, anti-inflammatory, and anticancer effects, making it a promising candidate for further investigation. These findings suggest that the binding affinity of quercetin and its potential functional relevance warrant deeper exploration in subsequent studies. Moreover, previous studies have also reported quercetin interacting with key target proteins involved in different cancers, supporting its anticancer mechanism, similar to the findings of the present study. For example, quercetin has been shown to bind AKT1 through hydrogen bonds with Val123 and Gly128 [62,63]. It interacts with PGR via hydrogen bonds with Ser247 and Tyr319 [63], with MMP9 through His123 and Glu402 [64], and with PTGS2 via Ser530 and Arg120 [65,66]. These interactions further support the role of quercetin in modulating critical cancer-related pathways.

Overall, the present study provided a comprehensive and systematic investigation of the anti-CRC effects and mechanisms of *E. sativa* by using network pharmacology, molecular docking, and MD simulation. The obtained results suggested that *E. sativa* could target multiple proteins and pathways involved in CRC, and that its active compounds could form stable and strong interactions with the key targets. The findings of this study may be able to contribute to a better understanding of the pharmacological mechanism by which *E. sativa* works against CRC, as well as supporting its potential development as a natural anticancer agent. While computational approaches such as molecular docking, molecular dynamics simulations, and network pharmacology provide valuable insights into the potential bioactivity of compounds, they have inherent limitations. Docking scores, though indicative of binding affinity, do not always correlate with actual biological efficacy due to factors such as solubility, metabolism, and bioavailability [67]. Similarly, molecular dynamics simulations rely on force field approximations that may not fully capture the complexity of protein–ligand interactions in a physiological environment [68]. Additionally, databases used for target prediction may contain biases or incomplete information, potentially affecting the accuracy of predicted interactions. Experimental validation, including in vitro and in vivo studies, is crucial to confirm the biological relevance of these computational findings and to overcome the constraints associated with in silico methods. However, to validate these computational predictions, further in vitro and in vivo studies are essential [69]. Experimental validation could involve assessing the cytotoxic effects of the selected compounds on CRC cell lines using MTT or Annexin V/PI assays. Additionally, western blot or RT-qPCR analyses could help determine their impact on the expression of relevant oncogenic and apoptotic markers [70]. Translating these promising in silico findings into preclinical and clinical settings involves several key steps. Preclinical studies in animal models would assess bioavailability, pharmacokinetics, and toxicity, while formulation strategies, such as nanoparticle-based delivery systems, would be explored to enhance stability and targeted delivery. If preclinical results are favorable, clinical trials would evaluate the safety, efficacy, and optimal dosage of *E. sativa* compounds in human subjects, potentially in combination with existing therapies [71]. Regulatory approval from agencies such as the FDA would be necessary before clinical use. Additionally, *E. sativa* compounds could be explored for their synergy with current treatments, potentially enhancing therapeutic efficacy or overcoming drug resistance. These efforts will be critical to advancing *E. sativa* from laboratory discoveries to clinical applications [72].

Moreover, *E. sativa* has been recognized for its nutritional benefits and potential therapeutic properties. However, its safety profile and potential toxicity in human models require careful consideration. The primary bioactive compounds in *E. sativa*, including glucosinolates, flavonoids, and phenolic compounds, contribute to its health benefits but may also pose risks under certain conditions. The presence of certain compounds, such as isothiocyanates, can lead to adverse effects if consumed in excessive amounts. For example, while these compounds have demonstrated anticancer properties, they may also exhibit cytotoxic effects on normal cells at high concentrations [73]. Additionally, the potential for allergic reactions to *E. sativa* should not be overlooked. Some individuals may experience hypersensitivity to cruciferous vegetables, leading to gastrointestinal disturbances or other allergic responses [12]. Furthermore, the presence of glucosinolates, while beneficial in moderate amounts, can lead to thyroid dysfunction if consumed excessively, particularly in individuals with pre-existing thyroid conditions [74].

## 4. Materials and Methods

### 4.1. A Virtual Screening of Active Ingredients

The investigation into the active bioactive compounds of *E. sativa* involved a comprehensive review of pertinent literature, and the IMPPAT database was utilized. The PubChem Explore Chemistry program was used to obtain the SMILES (Simplified Molecular-Input Line-Entry System) and structure of each active compound [75]. Subsequently, a virtual screening process was employed for all constituents of *E. sativa*, incorporating oral bioavailability (OB) and drug-likeness (DL). These parameters play a pivotal role in defining the absorption, distribution, metabolism, and excretion (ADME) properties of drugs. The criteria for inclusion for the study necessitated that the compound exhibit DL values higher than or equal to 0.18 and OB values exceeding 30%, ensuring adherence to ADME standards. Any biologically active compounds failing to meet these conditions were systematically excluded from further investigation. To compute DL and OB, Molsoft [76] and SwissADME [77] were utilized, contributing to the comprehensive evaluation of the selected active constituents.

### 4.2. Screening of Target Genes

The SwissTarget Prediction server [http://www.swisstargetprediction.ch/, accessed on 5 February 2024] was used for identifying potential target genes associated with the active constituents screened. In order to gain a deeper understanding of the molecular mechanisms underpinning the therapeutic effects of the herb on CRC, the next step consisted of predicting the genes that are involved in diseases. This was accomplished through the exploration of three databases: OMIM [https://www.omim.org/, accessed on 6 February 2024], GeneCards [https://www.genecards.org/, accessed on 6 February 2024], and DisGeNET [http://www.disgenet.org/, accessed on 6 February 2023]. The search was conducted using the keyword ‘Colorectal Cancer’ to retrieve genes associated with the disease. For DisGeNET, a cutoff criterion of “score gda > 0.1” was applied, while a score exceeding 30 was employed for GeneCards. DisGeNET serves as a versatile data system encompassing information on genes, disorders, and associated empirical studies. On the other hand, OMIM is an Online Mendelian Inheritance in Man database, offering insights into Mendelian disorders and over 16,000 genes, with a focus on the interplay between phenotype and genotype. The GeneCards database provides comprehensive information on the genome, proteome, and transcriptomes of organisms. In the following step, the data was analyzed using the FunRich tool to determine whether there was an overlap of genes between the predicted target genes of the screened compounds and the disease-related targets [78]. This approach facilitated the identification of common targets shared between active constituents and the specific disease under consideration, laying the groundwork for subsequent analytical processes.

### 4.3. Construction of Network

As a first step in elucidating the mechanism of action of *E. sativa* on colorectal cancer, we conducted a network analysis. This biomolecular interaction network was constructed, visualized, and analyzed using Cytoscape 3.10.1, a free software application with a graphical user interface that enables the importing, visual exploration, and analysis of biomolecular interaction networks [79]. Using this representation, nodes were used to illustrate the active constituents in the network, as well as the target genes within the network, and edges were used to indicate how the active constituents and the target genes are interconnected. As a result of utilizing the Network Analyzer tool within Cytoscape, topological properties such as degree, betweenness, subgroup, and closeness were calculated, which revealed the significance of the compound–target gene–pathway relationships within the network diagram. Moreover, the target genes exhibiting the highest degree of connectivity within the network were recognized as ‘key targets’ after identifying pivotal elements within the network. Based on the comprehensive network analysis provided by this study, valuable insights were provided into the intricate interactions that underlie the mechanism of *E. sativa* as it relates to colorectal cancer prevention.

### 4.4. Pathway and Functional Enrichment Analysis

In order to analyze gene enrichment along with KEGG pathway analysis, we used the Database for Annotation, Visualization, and Integrated Discovery (DAVID) [80]. There were three distinct levels at which functional annotation was performed on the identified key genes: cellular components (CC), molecular functions (MF), and biological processes (BP), which were all submitted to DAVID for functional annotation. The *p*-value ≥ 0.01 was applied in selecting the top 10 GO enrichments and the top 30 KEGG pathways for further analysis in this study.

### 4.5. PPI Network Construction and Molecular Docking Analysis

A PPI network was constructed from the protein–protein interactions (PPIs) data in the STRING database by selecting interactions with a confidence score of >0.4. Following this, these genes were uploaded to a database in order to construct an integrated PPI network [81].

This was followed by the importation of the obtained PPI network into Cytoscape software. In order to identify core regulatory genes within the PPI network and identify key targets within the network, the CytoHubba plugin within Cytoscape was employed. In order to evaluate the co-expression patterns of predicted key targets, the STRING database was used. As a follow-up to the results of network pharmacology, the X-ray 3D crystal structure of the targets was acquired from RCSB PDB in order to validate the results of network pharmacology [82]. The protein structures were prepared by removing bound ligands, water molecules, and heteroatoms, followed by the addition of polar hydrogen atoms. The docking grid box was centered on the active site of the protein, and the size was adjusted to accommodate the ligand’s binding site. The grid spacing was set to 1.0 Å, with the default docking parameters for AutoDock Vina, which include a maximum of 10 docked poses for each ligand. The ligands, including the compounds from *E. sativa* and the standard drug Camptosar, were optimized using the 3D structure and converted into the .pdbqt format using AutoDockTools. Each ligand’s rotatable bonds were defined for flexible docking, while the protein was treated as rigid. Docking analysis was performed with Autodock version 1.5.7 [83], and the active site was identified with information obtained from the Computed Atlas of Surface Topography of Proteins (CASTp) server (Table 1). In order to identify potential binding sites for the ligands, the docking scores for each ligand were used to filter the results during the screening process. The docked protein–ligand complexes have been extensively studied to understand the interactions formed between proteins and ligands during binding. In order to perform the subsequent analysis, it was decided to select the best-docked scores, indicating the lowest binding energy. A number of visualization tools were employed in the process of visualizing interactions between active compounds and their predicted targets using PyMOL and Discovery Studio Visualizer [84]. By using this approach, it was possible to obtain a more comprehensive understanding of the molecular interactions between proteins and their ligands.

### 4.6. Molecular Dynamics Simulation

MD analysis was conducted in order to investigate the conformational stability of ligands within receptor-binding pockets over the course of time. The CHARMM36m force field was used in the MD simulations and Gromacs version 2019.4 was used to run the simulations. In order to generate the force field parameters for the ligands, the SwissParam server was used to obtain the structure of the ligand topology. In order to minimize the vacuum in the system, the steepest descent algorithm was applied over 50,000 steps. The protein–ligand complex was enclosed by a cubic periodic box with dimensions of 0.5 nm in a water model based on TIP3P with dimensions of 0.5 nm. The system was neutralized by adding Na^+^ and Cl^−^ counterions to reach a salt concentration of 0.15 m. Following this, a series of equilibration calculations were performed, including NVT (constant number, volume, and temperature) and NPT (constant number, pressure, and temperature), which were carried out using the leap-frog algorithm over the course of 100 ps. After that, solvated protein–ligand complexes were subjected to a 100 ns run. In order to analyze the MD simulation data, the trajectory file has been removed from its periodic boundary conditions, and Chimera has been used to perform the analysis. In order to generate the graphs, XMGRACE (http://plasma-gate.weizmann.ac.il/Grace/, accessed on 16 February 2024) was used. By using this comprehensive approach, it was possible to examine the conformational dynamics of the ligand within receptor binding pockets in greater detail, as well as visualize the results obtained from MD [85].

## 5. Conclusions

The present integrated approach utilizing network pharmacology, molecular docking, and molecular dynamics simulation provided a comprehensive understanding of the anti-colorectal cancer effects and underlying mechanisms of *E. sativa*. Through the identification of six active compounds and 40 potential targets, construction of compound–target networks, and enrichment analysis, we elucidated the intricate modulation of multiple targets and pathways related to CRC by *E. sativa*. Notably, our findings revealed the anticancer properties of *E. sativa* due to the presence of food-bioactive compounds such as beta-ionone, 1-octanol, isorhamnetin, 2-hexenal, propionic acid, and quercetin. Molecular docking highlighted the favorable binding interactions between selected compounds (beta-ionone, 1-octanol, isorhamnetin, 2-hexenal, propionic acid, and quercetin) and key targets (AKT1, PGR, MMP9, and PTGS2), with quercetin and isorhamnetin exhibiting the highest binding affinity. Our study contributes valuable insights into the pharmacological mechanisms of *E. sativa* against CRC, suggesting its potential as a natural anticancer agent. However, the translation of these findings into clinical applications necessitates further experimental validation and clinical trials to affirm the efficacy and safety of *E. sativa* and its compounds in CRC treatment. The integration of computational approaches with experimental validations provides a solid foundation for advancing our understanding of *E. sativa* potential as a therapeutic agent against colorectal cancer. Future studies should focus on validating *E. sativa* phytoconstituents through in vitro and in vivo experiments, assessing their cytotoxicity, molecular mechanisms, and pharmacokinetics. Formulation strategies like nanoparticle-based delivery and small-scale clinical trials could enhance their therapeutic potential. Multi-omics approaches may further elucidate their role in colorectal cancer treatment.

## Figures and Tables

**Table 1:** Grid box coordinates and size parameters used in AutoDock Vina.

Sr.No.	Protein-Compound	Grid Size			Grid Centre			Grid Space
		**X**	**Y**	**Z**	**X**	**Y**	**Z**	
1	EGFR	40	40	40	−1.672	−48.365	18.337	0.375 Å
2	AKT1	40	44	40	9.511	−7.594	11.38	0.375 Å
3	SRC	44	40	40	12.833	−36.083	−5.417	0.375 Å
4	ESR1	36	30	36	30.153	−0.06	24.412	0.375 Å
5	PARP1	40	40	40	−37.474	6.782	−7.052	0.375 Å
6	MMP9	40	40	40	67.77	31.04	115.898	0.375 Å
7	PTGS2	40	40	40	24.507	50.313	20.363	0.375 Å
8	GSK3B	40	40	40	34.101	7.396	32.472	0.375 Å
9	PGR	40	40	40	27.176	13.149	63.279	0.375 Å
10	KDR	40	40	40	19.275	7.327	11.715	0.375 Å

## Data Availability

All data generated or analyzed during this study are included in this article.

## Supplementary Materials

The following supporting information can be downloaded at: https://www.mdpi.com/article/10.3390/ph18040453/s1, Table S1: Compound details of *E. sativa* plant extract IMPPAT data with PubChem ID, molecular weight, molecular formula, drug likeness, bioavailability and canonical SMILES. Table S2: Topological parameter analysis of *E. sativa* compounds (Betweenness: Indicates a node’s role as a bridge in the network. Higher values suggest key regulatory proteins; Closeness: Reflects how quickly a node connects to others. Higher values indicate central proteins; Subgraph: Measures a node’s involvement in multiple sub-networks, highlighting multifunctional proteins). Table S3: Topological parameter analysis of identified common genes between *E. sativa* compound and colorectal cancer (Betweenness: Indicates a node’s role as a bridge in the network. Higher values suggest key regulatory proteins; Closeness: Reflects how quickly a node connects to others. Higher values indicate central proteins; Subgraph: Measures a node’s involvement in multiple sub-networks, highlighting multifunctional proteins). Table S4: Interaction analysis of *E. sativa* compound against identified top ten hub genes.
