# Supplementary material for: Insights into the Therapeutic Targets and Molecular Mechanisms of Eruca sativa Against Colorectal Cancer: An Integrated Approach Combining Network Pharmacology, Molecular Docking and Dynamics Simulation
Source: Pharmaceuticals (Basel). 2025 Mar 24;18(4):453. doi: 10.3390/ph18040453 (PMC12030293; doi:10.3390/ph18040453)
Supplement: Supplementary file 1 [file pharmaceuticals-18-00453-s001.zip › pharmaceuticals-3485524-supplementary.pdf]

Table S1. Compound details of *E. sativa* plant extract IMPPAT data with PubChem ID, Molecular weight, Molecular Formula, Drug likeness, Bioavailability and Canonical SMILES.

Sr. No.	Compound	Chemical Structure	PubChem ID	Molecular Formula	Molecular Weight (g/mol)	Drug likeliness	Bioavailability Score	Canonical SMILES
1	(2R,3S,4S,5R,6S)-2-(hydroxymethyl)-6-(4-sulfanylbutylsulfanyl)oxane-3,4,5-triol	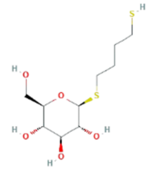	101204867	$C_{10}H_{20}O_5S_2$	284.4	-0.55	0.55	C(CCSC1C(C(C(C(O1)CO)O)O)O)CS
2	1-Eicosanol	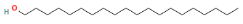	12404	$C_{20}H_{42}O$	298.5	-0.92	0.55	CCCCCCCCCCCCCCCCCCCCCO
3	1-Hexadecanol	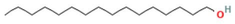	2682	$C_{16}H_{34}O$	242.44	-0.92	0.55	CCCCCCCCCCCCCCCCO
4	1-Isothiocyanato-4-methylpentane	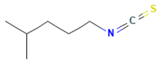	519452	$C_7H_{13}NS$	143.25	-1.22	0.55	CC(C)CCCN=C=S
5	1-Octanol	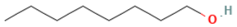	957	$C_8H_{18}O$	130.229	0.29	0.55	CCCCCCCCO
6	1-Octen-3-OL	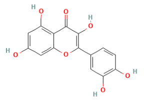	18827	$C_8H_{16}O$	128.21	-1.31	0.55	CCCCCC(C=C)O
7	2-Acetylthiazole	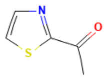	520108	C_5H_5NOS	127.17	-1.37	0.55	CC(=O)C1=NC=CS1
8	2-Hexen-1-OL	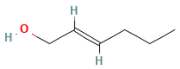	5318042	$C_6H_{12}O$	100.09	-1.34	0.55	CCCC=CCO
9	2-Hexenal	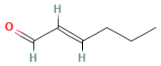	5281168	$C_6H_{10}O$	98.14	0.29	0.55	CCC/C=C/C=O
10	2-Isopropyl-3-methoxypyrazine	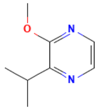	33166	$C_8H_{12}N_2O$	152.19	-1.47	0.55	CC(C)C1=NC=CN=C1OC
11	2-Methylanisole	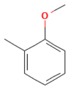	33637	$C_8H_{10}O$	122.16	-1.34	0.55	CC1=CC=CC=C1OC

12	2-Methylbutanoic acid	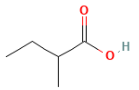	8314	$C_5H_{10}O_2$	102.13	-1.03	0.85	CCC(C)C(=O)O
13	2-Octanol	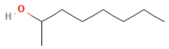	20083	$C_8H_{18}O$	130.229	-1.16	0.55	CCCCCCC(C)O
14	2-Pentenenitrile	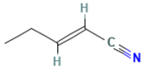	5364360	C_5H_7N	81.12	-1.35	0.55	CC/C=C/C#N
15	2-Pentylfuran	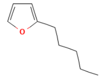	19602	$C_9H_{14}O$	138.21	-1.22	0.55	CCCCCC1=CC=CO1
16	3-Octanol	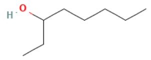	11527	$C_8H_{18}O$	130.229	-1.31	0.55	CCCCCC(CC)O
17	3-Octanone	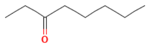	246728	$C_8H_{16}O$	128.21	-1.25	0.55	CCCCCC(=O)CC
18	4-(Methylthio)butyl thiocyanate	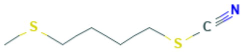	89295500	$C_6H_{11}NS_2$	161.3	-0.93	0.55	CSCCCCSC#N
19	4-Mercaptobutyl isothiocyanate	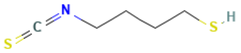	85704368	$C_5H_9NS_2$	147.3	-1.35	0.11	C(CCS)CN=C=S
20	4-Methylthiobutyl glucosinolate	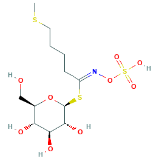	6537200	$C_{12}H_{23}NO_9S_3$	421.5	-0.51	0.55	CSCCCCC(=NOS(=O)(=O)O)SC1C(C(C(C(O1)CO)O)O)O
21	4-Pentenyl isothiocyanate	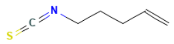	87436	C_6H_9NS	127.21	-1.32	0.55	C=CCCCN=C=S
22	5-(Methylsulfanyl)pentanenitrile	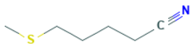	93320	$C_6H_{11}NS$	129.229	-0.91	0.55	CSCCCCC#N
23	5-Methylhexanenitrile	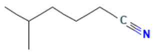	29593	$C_7H_{13}N$	111.18	-1.12	0.55	CC(C)CCCC#N
24	Acetophenone	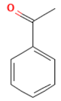	7410	C_8H_8O	120.15	-1.91	0.55	CC(=O)C1=CC=CC=C1
25	Allyl isothiocyanate	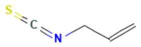	5971	C_4H_5NS	99.16	-1.44	0.11	C=CCN=C=S
26	alpha-Pinene	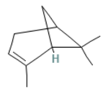	6654	$C_{10}H_{16}$	136.23	-1.45	0.55	CC1=CCC2CC1C2(C)C

27	Anethole	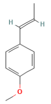	637563	$C_{10}H_{12}O$	148.2	-1.68	0.55	C/C=C/C1=CC=C(C=C1)OC
28	Benzaldehyde	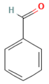	240	C_7H_6O	106.12	-1.81	0.55	C1=CC=C(C=C1)C=O
29	Berteroin	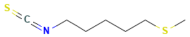	206037	$C_7H_{13}NS_2$	175.3	-1.06	0.55	CSCCCCCN=C=S
30	beta-Ionone	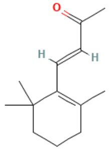	638014	$C_{13}H_{20}O$	192.3	0.33	0.55	CC1=C(C(CCC1)(C)C)/C=C/C(=O)C
31	Butyl isothiocyanate	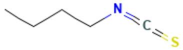	11613	C_5H_9NS	115.2	-1.68	0.55	CCCCN=C=S
32	cis-3-Hexen-1-ol	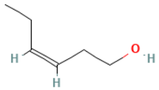	5281167	$C_6H_{12}O$	100.16	-1.15	0.55	CC/C=C\CCO
33	cis-3-Hexenyl 2-methylbutanoate	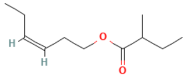	5365069	$C_{11}H_{20}O_2$	184.27	-0.87	0.55	CC/C=C\CCOC(=O)C(C)CC
34	cis-3-Hexenyl acetate	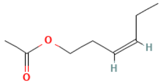	5363388	$C_8H_{14}O_2$	142.2	-1.07	0.55	CC/C=C\CCOC(=O)C
35	Decanoic acid	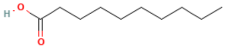	2969	$C_{10}H_{20}O_2$	172.26	-0.54	0.85	CCCCCCCCCC(=O)O
36	Dimethyl trisulfide	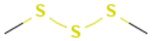	19310	$C_2H_6S_3$	126.3	-1.32	0.55	CSSSC
37	Docosane		12405	$C_{22}H_{46}$	310.6	-1.03	0.55	CCCCCCCCCCCCCCCCCCCCCC
38	Dodecane	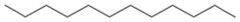	8182	$C_{12}H_{26}$	170.33	-1.03	0.55	CCCCCCCCCCCC
39	Eicosane		8222	$C_{20}H_{42}$	282.5	-1.03	0.55	CCCCCCCCCCCCCCCCCCCC
40	Erucic acid	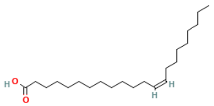	5281116	$C_{22}H_{42}O_2$	338.6	-0.3	0.85	CCCCCCCC/C=C\CCCCCCCCCCCC(=O)O
41	Erucin	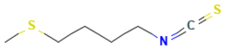	78160	$C_6H_{11}NS_2$	161.3	-1.06	0.55	CSCCCCN=C=S

42	Eugenol	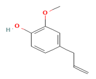	3314	$C_{10}H_{12}O_2$	164.2	-0.74	0.55	COC1=C(C=CC(=C1)CC=C)O
43	Furfural	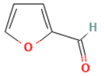	7362	$C_5H_4O_2$	96.08	-1.82	0.55	C1=COC(=C1)C=O
44	Furfuryl alcohol	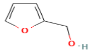	7361	$C_5H_6O_2$	98.1	-1.69	0.55	C1=COC(=C1)CO
45	Glucoerucin(1-)	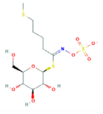	6537199	$C_{12}H_{22}NO_9S_3$	420.5	-0.51	0.11	CSCCCCC(=NOS(=O)(=O)[O-])SC1C(C(C(C(O1)CO)O)O)O
46	Glucoraphanin(1-)	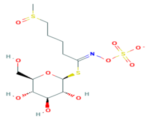	6325266	$C_{12}H_{22}NO_{10}S_3$	436.5	-0.66	0.55	CS(=O)CCCCC(=NOS(=O)(=O)[O-])SC1C(C(C(C(O1)CO)O)O)O
47	Heneicosane		12403	$C_{21}H_{44}$	296.6	-1.03	0.55	CCCCCCCCCCCCCCCCCCCCC
48	Hentriacontane		12410	$C_{31}H_{64}$	436.8	-1.03	0.55	CCCCCCCCCCCCCCCCCCCCCCCCCCCCCCC
49	Heptacosane		11636	$C_{27}H_{56}$	380.7	-1.03	0.55	CCCCCCCCCCCCCCCCCCCCCCCCCCC
50	Heptadecane	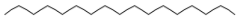	12398	$C_{17}H_{36}$	240.5	-1.03	0.55	CCCCCCCCCCCCCCCCC
51	Heptadecanoic acid	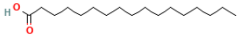	10465	$C_{17}H_{34}O_2$	270.5	-0.54	0.85	CCCCCCCCCCCCCCCCC(=O)O
52	Heptanal	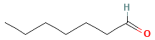	8130	$C_7H_{14}O$	114.19	-1.19	0.55	CCCCCCC=O
53	Heptanoic acid	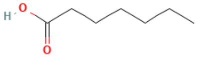	8094	$C_7H_{14}O_2$	130.18	-0.54	0.85	CCCCCCC(=O)O
54	Hexacosane		12407	$C_{26}H_{54}$	366.7	-1.03	0.55	CCCCCCCCCCCCCCCCCCCCCCCCCC
55	Hexadecane	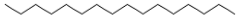	11006	$C_{16}H_{34}$	226.44	-1.03	0.55	CCCCCCCCCCCCCCCC
56	Hexanenitrile, 6-(methylthio)-	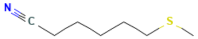	155939	$C_7H_{13}NS$	143.25	-0.91	0.55	CSCCCCCC#N
57	Hexanoic acid	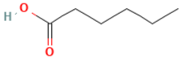	8892	$C_6H_{12}O_2$	116.16	-0.54	0.85	CCCCCC(=O)O
58	Hexyl isothiocyanate	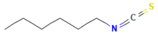	78120	$C_7H_{13}NS$	43.25	-1.56	0.55	CCCCCCN=C=S

59	Indole	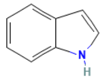	798	C_8H_7N	117.15	-2.75	0.55	C1=CC=C2C(=C1)C=CN2
60	Isophytol	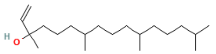	10453	$C_{20}H_{40}O$	296.5	-1.06	0.55	CC(C)CCCC(C)CCCC(C)CCCC(C)(C=C)O
61	Isorhamnetin	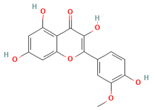	5281654	$C_{16}H_{12}O_7$	316.26	0.39	0.55	COC1=C(C=CC(=C1)C2=C(C(=O)C3=C(C=C(C=C3O2)O)O)O)O
62	Isorhamnetin 3-glucoside	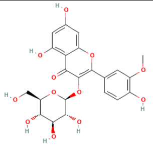	5318645	$C_{22}H_{22}O_{12}$	478.4	0.59	0.17	COC1=C(C=CC(=C1)C2=C(C(=O)C3=C(C=C(C=C3O2)O)O)OC4C(C(C(C(O4)CO)O)O)O)O
63	Lauric acid	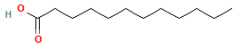	3893	$C_{12}H_{24}O_2$	200.32	-0.54	0.85	CCCCCCCCCCCC(=O)O
64	Linoleic acid	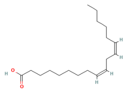	5280450	$C_{18}H_{32}O_2$	280.4	-0.30	0.55	CCCCC/C=C\C/C=C\CCCCCCCC(=O)O
65	Linolenic acid	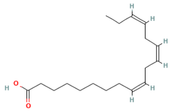	5280934	$C_{18}H_{30}O_2$	278.4	0.09	0.55	CC/C=C\C/C=C\C/C=C\CCCCCCCC(=O)O
66	Methyl decanoate	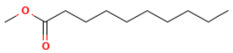	8050	$C_{11}H_{22}O_2$	186.29	-1.31	0.55	CCCCCCCCCC(=O)OC
67	Methyl linolenate	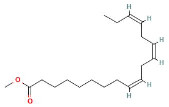	5319706	$C_{19}H_{32}O_2$	292.5	-0.93	0.55	CC/C=C\C/C=C\C/C=C\CCCCCCCC(=O)OC
68	Methyl palmitate	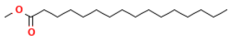	8181	$C_{17}H_{34}O_2$	270.5	-1.31	0.55	CCCCCCCCCCCCCCCC(=O)OC
69	Myristic acid	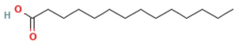	11005	$C_{14}H_{28}O_2$	228.37	-0.54	0.85	CCCCCCCCCCCCCC(=O)O
70	Nonacosane		12409	$C_{29}H_{60}$	408.8	-1.03	0.55	CCCCCCCCCCCCCCCCCCCCCCCCCCCCC
71	Nonadecane		12401	$C_{19}H_{40}$	268.5	-1.03	0.55	CCCCCCCCCCCCCCCCCCC
72	Nonanal	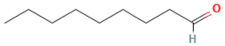	31289	$C_9H_{18}O$	142.24	-1.19	0.55	CCCCCCCCC=O
73	Nonanamide	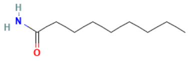	70709	$C_9H_{19}NO$	157.25	-0.77	0.55	CCCCCCCCC(=O)N
74	Nonane		8141	C_9H_{20}	128.25	-1.03	0.55	CCCCCCCCC

75	Nonanoic acid	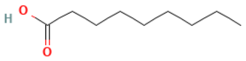	8158	$C_9H_{18}O_2$	158.24	-0.54	0.85	CCCCCCCCC(=O)O
76	Octacosane		12408	$C_{28}H_{58}$	394.8	-1.03	0.55	CCCCCCCCCCCCCCCCCCCCCCCCCCCC
77	Octadecane		11635	$C_{18}H_{38}$	254.5	-1.03	0.55	CCCCCCCCCCCCCCCCCC
78	Octanoic acid	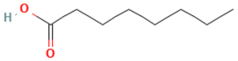	379	$C_8H_{16}O_2$	144.21	-0.54	0.85	CCCCCCCC(=O)O
79	Oleamide	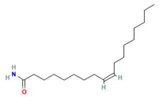	5283387	$C_{18}H_{35}NO$	281.5	-0.56	0.55	CCCCCCCC/C=C\CCCCCCCC(=O)N
80	Oleic acid	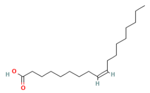	445639	$C_{18}H_{34}O_2$	282.5	-0.3	0.85	CCCCCCCC/C=C\CCCCCCCC(=O)O
81	Palmitic acid	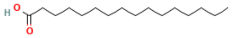	985	$C_{16}H_{32}O_2$	256.42	-0.54	0.85	CCCCCCCCCCCCCCCC(=O)O
82	Palmitoleic acid	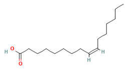	445638	$C_{16}H_{30}O_2$	254.41	-0.3	0.85	CCCCCC/C=C\CCCCCCCC(=O)O
83	Pentacosane		12406	$C_{25}H_{52}$	352.7	-1.03	0.55	CCCCCCCCCCCCCCCCCCCCCCCCC
84	Pentadecane	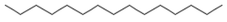	12391	$C_{15}H_{32}$	212.41	-1.03	0.55	CCCCCCCCCCCCCCC
85	Pentadecanoic acid	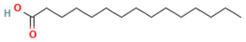	13849	$C_{15}H_{30}O_2$	242.4	-0.54	0.85	CCCCCCCCCCCCCCC(=O)O
86	Phenylacetaldehyde	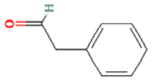	998	C_8H_8O	120.15	-1.71	0.55	C1=CC=C(C=C1)CC=O
87	Phytol	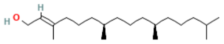	5280435	$C_{20}H_{40}O$	296.5	-0.87	0.55	CC(C)CCCC(C)CCCC(C)CCCC(=CCO)C
88	Propionic acid	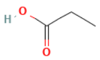	1032	$C_3H_6O_2$	74.08	0.29	0.85	CCC(=O)O
89	Quercetin	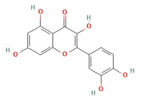	5280343	$C_{15}H_{10}O_7$	302.23	0.52	0.55	C1=CC(=C(C=C1C2=C(C(=O)C3=C(C=C(C=C3O2)O)O)O)O)O
90	Squalene	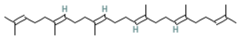	638072	$C_{30}H_{50}$	410.7	-0.90	0.55	CC(=CCC/C(=C/CC/C(=C/CC/C=C(/CC/C=C(/CCC=C(C)C)\C)\C)/C)/C)C

91	Stearic acid	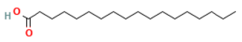	5281	$C_{18}H_{36}O_2$	284.5	-0.54	0.85	CCCCCCCCCCCCCCCCCC(=O)O
92	Sulforaphane	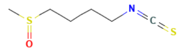	5350	$C_6H_{11}NOS_2$	177.3	-1.26	0.85	CS(=O)CCCCN=C=S
93	Tetracosane		12592	$C_{24}H_{50}$	338.7	-1.03	0.55	CCCCCCCCCCCCCCCCCCCCCCCC
94	Tetradecane	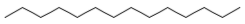	12389	$C_{14}H_{30}$	198.39	-1.03	0.55	CCCCCCCCCCCCCC
95	Tetrahydrothiophene	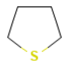	1127	C_4H_8S	88.17	-1.39	0.55	C1CCSC1
96	Thiophene	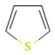	8030	C_4H_4S	84.14	-1.31	0.55	C1=CSC=C1
97	trans-2-Icosenoic acid	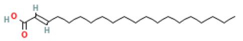	6438157	$C_{20}H_{38}O_2$	310.5	-1.04	0.55	CCCCCCCCCCCCCCCCC/C=C/C(=O)O
98	trans-3-Hexen-1-ol	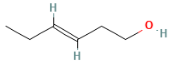	5284503	$C_6H_{12}O$	100.16	-1.15	0.55	CC/C=C/CCO
99	Triacontane		12535	$C_{30}H_{62}$	422.8	-1.03	0.55	CCCCCCCCCCCCCCCCCCCCCCCCCCCCCC
100	Tricosane		12534	$C_{23}H_{48}$	324.6	-1.03	0.55	CCCCCCCCCCCCCCCCCCCCCCC
101	Tridecane	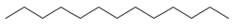	12388	$C_{13}H_{28}$	184.36	-1.03	0.55	CCCCCCCCCCCCC
102	Tridecanoic acid	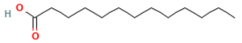	12530	$C_{13}H_{26}O_2$	214.34	-0.54	0.85	CCCCCCCCCCCCC(=O)O
103	Undecane	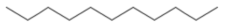	14257	$C_{11}H_{24}$	156.31	-1.03	0.55	CCCCCCCCCCC
104	Undecanoic acid	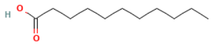	8180	$C_{11}H_{22}O_2$	186.29	-0.54	0.85	CCCCCCCCCCC(=O)O

Table S2. Topological parameter analysis of *E. sativa* compounds (Betweenness: Indicates a node's role as a bridge in the network. Higher values suggest key regulatory proteins; Closeness: Reflects how quickly a node connects to others. Higher values indicate central proteins; Subgraph: Measures a node's involvement in multiple sub-networks, highlighting multifunctional proteins).

Sr. No.	Compound	Degree	Betweenness	Closeness	Subgragh
1	Quercetin	20	558.7195	0.4787234	158.52
2	Isorhamnetin	20	558.7195	0.4787234	158.52
3	beta-Ionone	16	677.40344	0.4411765	59.5128
4	1-Octanol	11	563.4524	0.4166667	27.1155
5	2-Hexenal	10	393.7052	0.3947369	22.812
6	Propionic acid	2	88	0.2368421	2.24195

Table S3. Topological parameter analysis of identified common genes between *E. sativa* compound and colorectal cancer (Betweenness: Indicates a node's role as a bridge in the network. Higher values suggest key regulatory proteins; Closeness: Reflects how quickly a node connects to others. Higher values indicate central proteins; Subgraph: Measures a node's involvement in multiple sub-networks, highlighting multifunctional proteins).

Sr. No.	Gene	Degree	Betweenness	Closeness	Subgragh
1	EGFR	32	141.12471	0.8478261	26748474
2	AKT1	32	141.85869	0.8478261	26301706
3	ESR1	27	42.48465	0.7647059	23733096
4	SRC	27	70.82495	0.7647059	22640066
5	PARP1	25	101.313515	0.7358491	20158692
6	MMP9	25	80.665535	0.7222222	19702402
7	PTGS2	24	38.256866	0.7222222	19003864
8	GSK3B	23	24.424156	0.6964286	18117456
9	KDR	22	15.7471	0.6842105	17658582
10	IGF1R	21	13.9446945	0.6842105	17125370
11	PGR	22	24.552143	0.6964286	16634506
12	MMP2	20	7.9872727	0.6610169	16159863
13	AR	20	17.162622	0.6724138	14541996
14	MAP2K1	19	8.613773	0.6393443	14232814
15	MAPK1	21	51.06928	0.6724138	14208701
16	JAK2	18	5.3669477	0.6290323	13325194
17	MET	18	18.05653	0.6393443	12675299
18	PIK3R1	17	5.454452	0.6	11249581

19	TERT	16	3.984496	0.609375	10734611
20	ESR2	16	7.8063455	0.6190476	10457568
21	ABCB1	17	31.86955	0.6290323	9696604
22	CDK2	15	8.51282	0.6190476	8627739
23	ABCG2	16	28.050371	0.6190476	8454327
24	CYP19A1	15	106.91261	0.6190476	6249300
25	ALK	13	6.643694	0.5909091	6054906.5
26	PIK3CG	11	0.3509804	0.5492958	5515338.5
27	AURKA	10	2.004762	0.5492958	4095606.5
28	MMP1	9	1.8055556	0.5416667	3780005.2
29	TYMS	11	23.449879	0.5652174	2508416.8
30	PPARD	7	5.431502	0.5342466	1595120.6
31	CYP17A1	7	2.915751	0.527027	1510438.1
32	NOS1	5	0.2857143	0.5131579	914689.25
33	ABCC1	5	0	0.5131579	815598
34	EGLN1	3	0	0.4936709	577454
35	CYP27B1	6	11.175837	0.5131579	498934.75
36	PTPRF	3	0.14285715	0.4875	397271.94
37	CYP2A6	5	11.42674	0.4642857	232545.08
38	TYMP	3	2.3226395	0.4756098	107699.84
39	TDP2	1	0	0.4285714	51294.113
40	NQO2	1	0	0.3861386	15949.659

Table S4. Interaction analysis of *E. sativa* compound against identified top ten hub genes.

Sr. No.	Protein - Compound	Receptor - Ligand	Interaction Type	Distance
1	AKT1-Quercetin	A:ASN54:ND2 - UNL1:O	Conventional Hydrogen Bond	3.157
		UNL1:H - A:GLN79:OE1	Conventional Hydrogen Bond	1.995
		UNL1:H - A:THR211:O	Conventional Hydrogen Bond	2.853
		UNL1:H - A:ILE290:O	Conventional Hydrogen Bond	2.904
		A:ASP292:OD1 - UNL1	Pi-Anion	4.675
		A:LEU264:CD2 - UNL1	Pi-Sigma	3.901
		A:TRP80 - UNL1	Pi-Pi Stacked	3.838
		A:TRP80 - UNL1	Pi-Pi Stacked	4.543
		A:TRP80 - UNL1	Pi-Pi Stacked	4.975
		A:TRP80 - UNL1	Pi-Pi Stacked	5.733
		UNL1 - A:LEU210	Pi-Alkyl	4.488
		UNL1 - A:VAL270	Pi-Alkyl	3.983
2	PGR-Quercetin	A:ASN719:HD21 - UNL1:O	Conventional Hydrogen Bond	3.094
		A:ARG766:HH2 - UNL1:O	Conventional Hydrogen Bond	2.646
		UNL1:H - A:MET801:SD	Conventional Hydrogen Bond	2.643
		A:MET801:SD - UNL1	Pi-Sulfur	5.438
		A:PHE778 - UNL1	Pi-Pi T-shaped	5.003
		UNL1 - A:CYS891	Pi-Alkyl	5.186
		UNL1 - A:MET759	Pi-Alkyl	5.487
		UNL1 - A:LEU763	Pi-Alkyl	5.121
3	MMP9-Quercetin	UNL1:H - A:LEU418:O	Conventional Hydrogen Bond	2.552
		UNL1:H - A:TYR420:O	Conventional Hydrogen Bond	2.405
		UNL1:H - A:ARG424:O	Conventional Hydrogen Bond	2.758
		A:TYR423:CA - UNL1	Pi-Sigma	3.924
		A:HIS401 - UNL1	Pi-Pi Stacked	4.687
		UNL1 - A:LEU188	Pi-Alkyl	4.646
		UNL1 - A:VAL398	Pi-Alkyl	5.067
		UNL1 - A:LEU188	Pi-Alkyl	4.434
4	PTGS2-Quercetin	A:ASN43:HN - UNL1:O	Conventional Hydrogen Bond	2.630
		UNL1:H - A:ALA151:O	Conventional Hydrogen Bond	1.968
		UNL1:H - A:ASP125:OD2	Conventional Hydrogen Bond	2.122
		UNL1:H - A:GLU465:OE1	Conventional Hydrogen Bond	2.743
		UNL1 - A:LEU152	Pi-Alkyl	5.178
		UNL1 - A:LYS468	Pi-Alkyl	5.251
5	PARP1-Quercetin	UNL1:H - A:SER904:OG	Conventional Hydrogen Bond	2.137
		A:HIS862:CE1 - UNL1:O	Carbon Hydrogen Bond	3.397
		UNL1:H - A:TYR889	Pi-Donor Hydrogen Bond	2.569

		A:TYR907 - UNL1	Pi-Pi Stacked	3.672
		A:TYR907 - UNL1	Pi-Pi Stacked	4.564
		A:TYR896 - UNL1	Pi-Pi T-shaped	4.976
		UNL1 - A:ALA898	Pi-Alkyl	4.962
6	GSK3B-Quercetin	UNL1:H - A:ASN186:OD1	Conventional Hydrogen Bond	1.889
		UNL1:H - A:ASP200:OD2	Conventional Hydrogen Bond	2.722
		UNL1:H - A:VAL135:O	Conventional Hydrogen Bond	2.334
		A:ASP200:OD1 - UNL1	Pi-Anion	4.946
		A:ILE62:CD1 - UNL1	Pi-Sigma	3.943
		UNL1 - A:VAL70	Pi-Alkyl	4.539
		UNL1 - A:ALA83	Pi-Alkyl	5.008
		UNL1 - A:LEU188	Pi-Alkyl	4.722
		UNL1 - A:CYS199	Pi-Alkyl	4.600
		UNL1 - A:ALA83	Pi-Alkyl	4.485
		UNL1 - A:LEU188	Pi-Alkyl	4.676
		UNL1 - A:VAL70	Pi-Alkyl	5.259
		UNL1 - A:CYS199	Pi-Alkyl	5.191
7	SRC-Isorhamnetin	A:MET341:HN - UNL1:O	Conventional Hydrogen Bond	1.719
		UNL1:H - A:THR338:OG1	Conventional Hydrogen Bond	2.431
		A:LEU393:CD1 - UNL1	Pi-Sigma	3.873
		A:ALA403 - UNL1:C	Alkyl	3.696
		UNL1:C - A:MET314	Alkyl	4.931
		UNL1:C - A:VAL323	Alkyl	3.834
		UNL1 - A:LEU273	Pi-Alkyl	4.844
		UNL1 - A:VAL281	Pi-Alkyl	5.156
		UNL1 - A:VAL281	Pi-Alkyl	5.058
		UNL1 - A:ALA293	Pi-Alkyl	4.131
		UNL1 - A:LEU393	Pi-Alkyl	5.445
		UNL1 - A:LYS295	Pi-Alkyl	5.122
8	EGFR-Quercetin	UNL1:H - A:ALA743:O	Conventional Hydrogen Bond	2.600
		UNL1:H - A:THR790:OG1	Conventional Hydrogen Bond	2.746
		A:THR790:OG1 - UNL1	Pi-Donor Hydrogen Bond	3.719
		A:PHE723 - UNL1	Pi-Pi T-shaped	5.461
		A:PHE723 - UNL1	Pi-Pi T-shaped	5.794
		UNL1 - A:VAL726	Pi-Alkyl	4.375
		UNL1 - A:ALA743	Pi-Alkyl	4.710
		UNL1 - A:VAL726	Pi-Alkyl	4.919
		UNL1 - A:ALA743	Pi-Alkyl	4.517
		UNL1 - A:LYS745	Pi-Alkyl	4.206
		UNL1 - A:LEU718	Pi-Alkyl	4.692

		UNL1 - A:VAL726	Pi-Alkyl	4.871
9	ESR1-Quercetin	A:HIS524:HD1 - UNL1:O	Conventional Hydrogen Bond	2.183
		UNL1:H - A:GLU419:O	Conventional Hydrogen Bond	2.515
		UNL1:H - A:GLY420:O	Conventional Hydrogen Bond	2.615
		A:MET388:CA - UNL1:O	Carbon Hydrogen Bond	3.637
		UNL1 - A:LEU346	Pi-Alkyl	5.272
		UNL1 - A:MET388	Pi-Alkyl	5.366
		UNL1 - A:LEU346	Pi-Alkyl	5.086
		UNL1 - A:ALA350	Pi-Alkyl	4.749
		UNL1 - A:LEU387	Pi-Alkyl	4.838
		UNL1 - A:MET421	Pi-Alkyl	5.462
		UNL1 - A:LEU525	Pi-Alkyl	4.796
10	KDR-Quercetin	UNL1:H - A:ILE1044:O	Conventional Hydrogen Bond	2.879
		A:LYS868:NZ - UNL1	Pi-Cation	4.312
		A:LEU889:CD2 - UNL1	Pi-Sigma	3.588
		A:VAL916:CG2 - UNL1	Pi-Sigma	3.942
		A:VAL916:CG2 - UNL1	Pi-Sigma	3.922
		A:CYS1045:SG - UNL1	Pi-Sulfur	5.607
		A:CYS1045:SG - UNL1	Pi-Sulfur	5.006
		UNL1 - A:VAL899	Pi-Alkyl	4.903
		UNL1 - A:VAL848	Pi-Alkyl	4.634
		UNL1 - A:ALA866	Pi-Alkyl	4.408
		UNL1 - A:VAL899	Pi-Alkyl	5.469
